# Diagnostic and clinical relevance of the autophago-lysosomal network in human gliomas

**DOI:** 10.18632/oncotarget.7910

**Published:** 2016-03-04

**Authors:** Lukas Jennewein, Michael W. Ronellenfitsch, Patrick Antonietti, Elena I. Ilina, Jennifer Jung, Daniela Stadel, Lisa-Marie Flohr, Jenny Zinke, Janusz von Renesse, Ulrich Drott, Peter Baumgarten, Anne K. Braczynski, Cornelia Penski, Michael C. Burger, Jean-Philippe Theurillat, Joachim P. Steinbach, Karl-Heinz Plate, Ivan Dikic, Simone Fulda, Christian Brandts, Donat Kögel, Christian Behrends, Patrick N. Harter, Michel Mittelbronn

**Affiliations:** ^1^ Neurological Institute (Edinger Institute), Goethe University, Frankfurt am Main, Germany; ^2^ Senckenberg Institute of Neurooncology, Goethe University, Frankfurt am Main, Germany; ^3^ German Cancer Consortium (DKTK) and German Cancer Research Center (DKFZ), Heidelberg, Germany; ^4^ Experimental Neurosurgery, Department of Neurosurgery, Goethe University, Frankfurt am Main, Germany; ^5^ Institute of Biochemistry II, Goethe University, Frankfurt am Main, Germany; ^6^ Department of Neurosurgery, Goethe University, Frankfurt am Main, Germany; ^7^ Institute of Cell Biology, ETH Zürich, Switzerland; ^8^ Institute for Experimental Cancer Research in Pediatrics, Goethe University, Frankfurt am Main, Germany; ^9^ Department of Medicine, Hematology/Oncology, Goethe University, Frankfurt am Main, Germany

**Keywords:** astrocytoma, glioblastoma, autophagy, apoptosis, LC3B

## Abstract

Recently, the conserved intracellular digestion mechanism ‘autophagy’ has been considered to be involved in early tumorigenesis and its blockade proposed as an alternative treatment approach. However, there is an ongoing debate about whether blocking autophagy has positive or negative effects in tumor cells. Since there is only poor data about the clinico-pathological relevance of autophagy in gliomas *in vivo*, we first established a cell culture based platform for the *in vivo* detection of the autophago-lysosomal components. We then investigated key autophagosomal (LC3B, p62, BAG3, Beclin1) and lysosomal (CTSB, LAMP2) molecules in 350 gliomas using immunohistochemistry, immunofluorescence, immunoblotting and qPCR. Autophagy was induced pharmacologically or by altering oxygen and nutrient levels. Our results show that autophagy is enhanced in astrocytomas as compared to normal CNS tissue, but largely independent from the WHO grade and patient survival. A strong upregulation of LC3B, p62, LAMP2 and CTSB was detected in perinecrotic areas in glioblastomas suggesting micro-environmental changes as a driver of autophagy induction in gliomas. Furthermore, glucose restriction induced autophagy in a concentration-dependent manner while hypoxia or amino acid starvation had considerably lesser effects. Apoptosis and autophagy were separately induced in glioma cells both *in vitro* and *in vivo*. In conclusion, our findings indicate that autophagy in gliomas is rather driven by micro-environmental changes than by primary glioma-intrinsic features thus challenging the concept of exploitation of the autophago-lysosomal network (ALN) as a treatment approach in gliomas.

## INTRODUCTION

Glioblastoma (GBM) WHO grade IV is the most common primary brain tumor exhibiting mean overall survival of approximately 12 months despite aggressive treatment [1–3]. GBM often harbor *TP53* mutations leading to impaired apoptosis [4, 5] or alterations of the AKT/mTOR pathway as a consequence of *PTEN* mutation [6]. Autophagy is suppressed by the AKT/mTOR pathway activation constituting a highly conserved digestion mechanism for protein aggregates and dysfunctional organelles to regain energy by recycling amino acids in malnutritive conditions like starvation or hypoxia [7, 8]. Autophagy is also considered a cancer-promoting mechanism conferring therapy- and starvation-resistance to tumor cells including gliomas [9, 10, 11, 12]. Previously, autophagy was proposed as an alternative cell death mechanism (type-II cell death) to apoptosis (cell death type I) [13]. There is an ongoing controversial discussion on whether the inhibition or the induction of autophagy could be exploited as a new anti-cancer treatment and how autophagy-targeting drugs might be applied within the standard radio-chemotherapeutic therapy regimens in cancer patients [14]. Even though there are already ongoing phase I/II clinical trials investigating autophagy-targeting drugs in glioma patients [15], the definite role of autophagy and the question whether autophagy is a promising adjuvant therapeutic target in gliomas remains unclear. A major problem in monitoring autophagy is that alterations of the markers LC3B and p62 can result from either autophagy induction or blockade of the autophagic flux [16]. To elucidate this cellular digestion process in gliomas *in vivo*, we established a cell culture based platform for the morphological on-slide detection of autophagy. Previously, autophagy activation was almost exclusively assessed by LC3B-I to -II conversion in immunoblotting [17]. We analyzed 350 astrocytomas for autophagy-associated markers LC3B, p62, BAG3, Beclin1 and the lysosomal markers LAMP2 and Cathepsin B (CTSB) to investigate whether also the lysosomal machinery is altered. We aimed at defining the patterns of autophagy induction in human astrocytomas WHO grade I to IV and to re-assess autophagy and its clinico-pathological relevance in glioma biology. This data should contribute to a better understanding of the autophago-lysosomal system in gliomas and provide a reference point for following studies discussing autophagy as a tumor promoting or suppressing mechanism and potential glioma therapy target.

## RESULTS

### Methodological platform to monitor the autophago-lysosomal network *in vivo*

**Figure 1:**
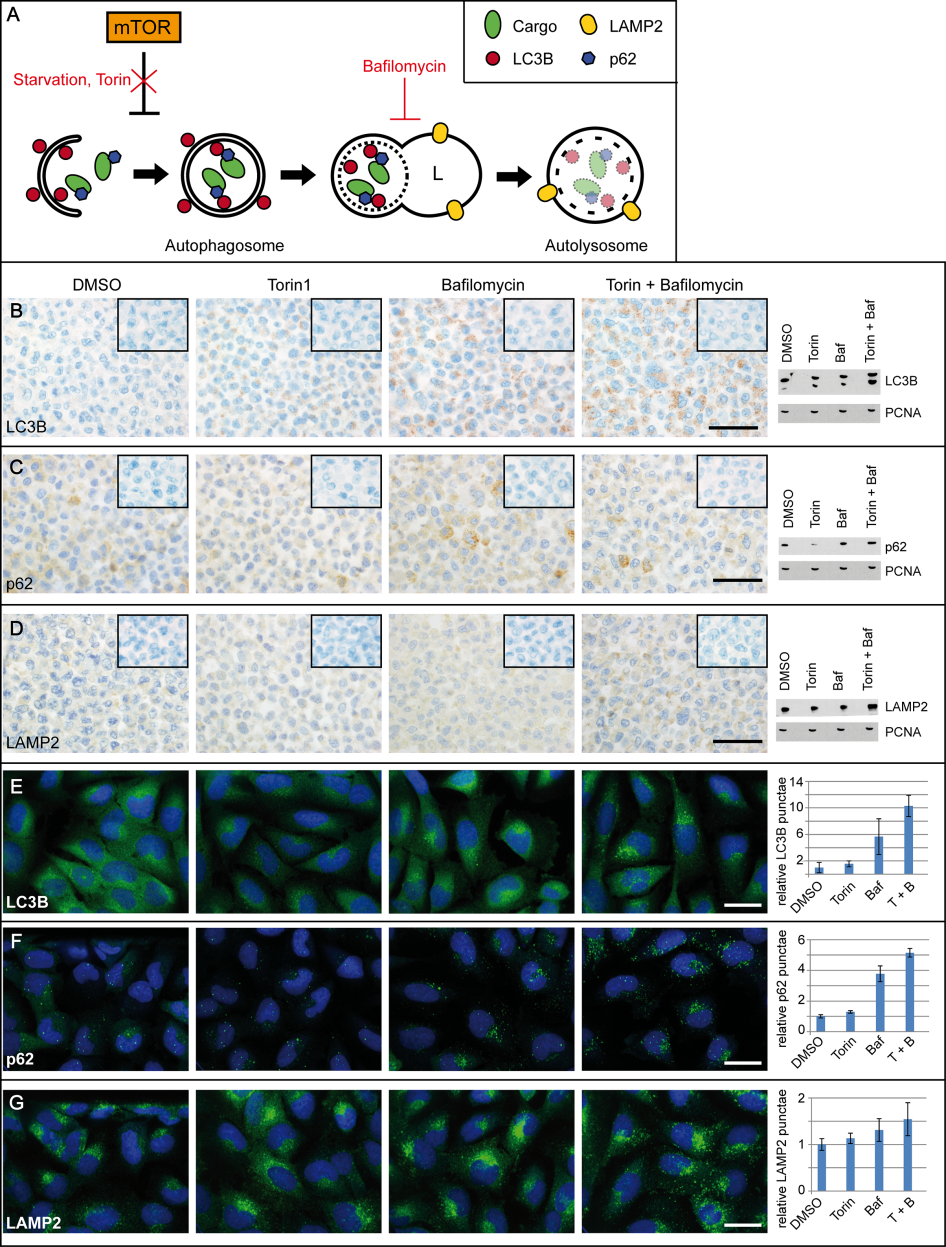
Platform for the immunocytochemical analyses of key autophago-lysosomal markers (**A**) Schematic overview of the autophago-lysosomal pathway including modulators of the induction of autophagy as well as inhibitors of the autophagic flux used in Figure 1B–1G. (**B–D**) Immunohistochemistry with antibodies against (B) LC3B, (C) p62 and (D) LAMP2 in LNT-229 glioma cells treated with DMSO as control condition, the activators of autophagosome formation Torin1 (250 nM) or BafA1 (100 nM) as well as the combination of both Torin1 and BafA1 (treatment time 2 h in each condition). Negative controls of the immunocytochemical stainings are shown in the right upper corner of each picture. Scale bars: 50 μm. Corresponding immunoblots were performed from each treatment condition. (**E–G**) LNT-229 glioma cells were treated with Torin1 (2 h, 250 nM) and/or BafA1 (2 h, 100 nM), fixed and labeled with (E) anti-LC3B, (F) anti-p62 or (G) anti-LAMP2 antibodies, respectively. DRAQ5 was used to stain nuclei. Scale bars: 10 μm. Images were acquired with Opera High Content Screening System and antibody-labeled spots were automatically counted using Acapella software. Data represent mean of quadruplicates ± standard deviation, normalized to DMSO control.

To date, autophagy induction is mainly assessed using immunoblotting to detect the conversion of LC3B-I to LC3B-II. Alternatively, artificial GFP-LC3B fusion proteins in cell culture models are used to trace autophagy [18]. To evaluate antibody suitability, we treated LNT-229 cells with Torin1 to induce autophagy via inhibition of mTOR and Bafilomycin A1 (BafA1) to inhibit autolysosomal acidification, thus blocking the autophagic flux (Figure 1A). Immunocytochemical stainings correlated with immunoblotting results (Figure 1B–1D) Immunohistochemistry showed more LC3B-positive punctae and immunoblotting revealed an increase in LC3B-II levels when cells were treated with Torin1. This effect was further increased upon BafA1 and combined Torin1/BafA1 treatment (Figure 1B). BafA1 and Torin1 treated LNT-229 showed a prominent LC3B conversion (Figure 1B) and increased LC3B punctae formation in immunofluorescent stainings (Figure 1E). P62 levels decreased upon Torin1 treatment but increased after addition of BafA1 in immunohistochemistry, western blot and immunofluorescence. (Figure 1C, 1F). LAMP2 levels remained almost constant in LNT-229 cells treated with Torin1 or BafA1 alone but increased when combining the two substances (Figure 1D). Immunofluorescent analyses of LAMP2-positive punctae displayed a slight enhancement under Torin1 and BafA1 treatment (Figure 1G). Comparisons between immunocytochemistry and immunoblot for Beclin1 (Supplementary Figure S1A), BAG3 (Supplementary Figure S1B) and Cathepsin B (CTSB) (Supplementary Figure S1C) are provided in Supplementary Figure S1, displaying no considerable changes upon Torin and/or Bafilomycin treatment except for a decrease in immunocytochemical expression intensity of BAG3 upon treatment. We further performed starvation experiments with glioma cell lines with and without p53 mutation to investigate potential differences in the activation of the autophago-lysosomal pathway (ALP) in relation to the p53 mutation status. The glioma cell line U87 (p53 wildtype) showed increased basal expression levels of LC3B and p62 compared to LNT-229 (p53 wildtype), and the p53 mutated glioma cell lines T98G and MZ-18 (Supplementary Figure S2). Nutrient restriction experiments showed no overall differences in ALP marker expression when comparing p53 mutated and p53 wildtype glioma cell lines (Supplementary Figure S3–S5). Antibody specificity was corroborated by the analyses of inducible HA-tagged LC3B (Supplementary Figure S6A–S6C) and p62 (Supplementary Figure S6D–S6F) as well as knockdown and overexpression experiments for BAG3 (data not shown). These findings indicate that our immunocytochemical setup is able to reflect different levels of autophagy induction on a cellular level.

### Autophagy is enhanced in human gliomas as compared to normal brain but independent from the grade of malignancy and patient survival

**Figure 2:**
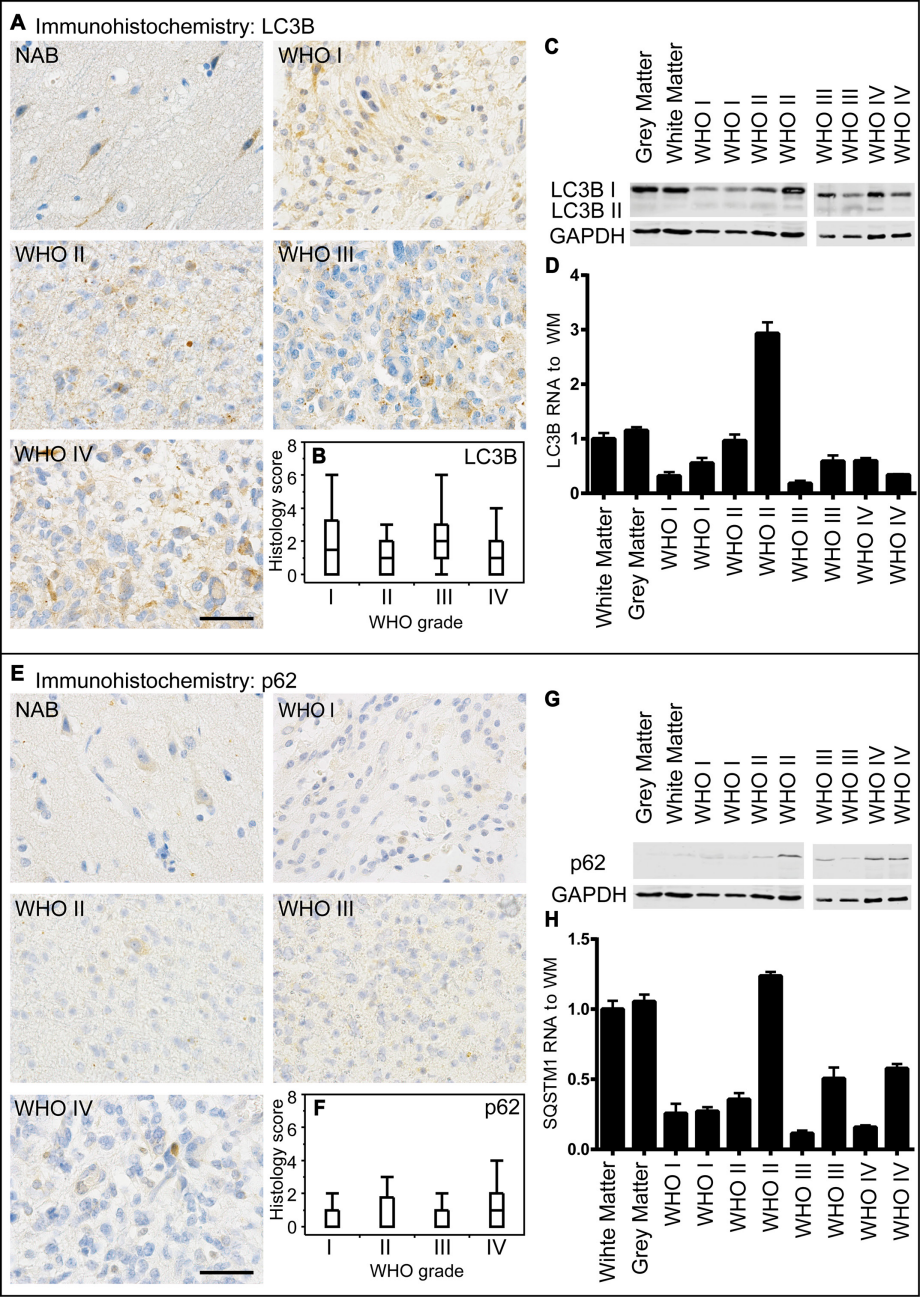
LC3B and p62 expression in human astrocytomas (**A**) Representative immunohistochemical staining against LC3B of normal appearing brain (NAB) tissue, pilocytic astrocytoma WHO grade I, diffuse astrocytoma WHO grade II, anaplastic astrocytoma WHO grade III and glioblastoma WHO grade IV. Scale bar: 50 μm. (**B**) Box blots showing LC3B scores (frequency × intensity) in 46 pilocytic astrocytomas WHO grade I (min: 0, max: 12, median: 1.5), 14 diffuse astrocytomas WHO grade II (min: 0, max: 6, median: 1), 33 anaplastic astrocytomas WHO grade III (min: 0, max: 8, median: 2) and 249 glioblastomas WHO grade IV (min: 0, max: 9, median: 1). (**C**) LC3B immunoblotting of normal grey matter, normal white matter and two astrocytomas of each WHO grade. (**D**) Relative LC3B mRNA levels of normal grey and white matter and two astrocytomas of each WHO grade normalized to normal white matter assessed by qPCR. (**E**) Representative immuno-histochemical staining against p62 of NAB, pilocytic astrocytoma WHO grade I, diffuse astrocytoma WHO grade II, anaplastic astrocytoma WHO grade III and glioblastoma WHO grade IV. Scale bar: 50 μm. (**F**) Box blots showing p62 scores (frequency × intensity) in 46 pilocytic astrocytomas WHO grade I (min: 0, max: 3, median: 0), 16 diffuse astrocytomas WHO grade II (min: 0, max: 3, median: 0), 34 anaplastic astrocytomas WHO grade III (min: 0, max: 3, median: 0) and 252 glioblastomas WHO grade IV (min: 0, max: 9, median: 1) (**G**) p62 immunoblotting of normal grey and white matter, and two astrocytomas of each WHO grade. (**H**) Relative p62 mRNA levels of normal and white matter and two astrocytomas of each WHO grade normalized to normal white matter assessed by qPCR.

**Figure 3:**
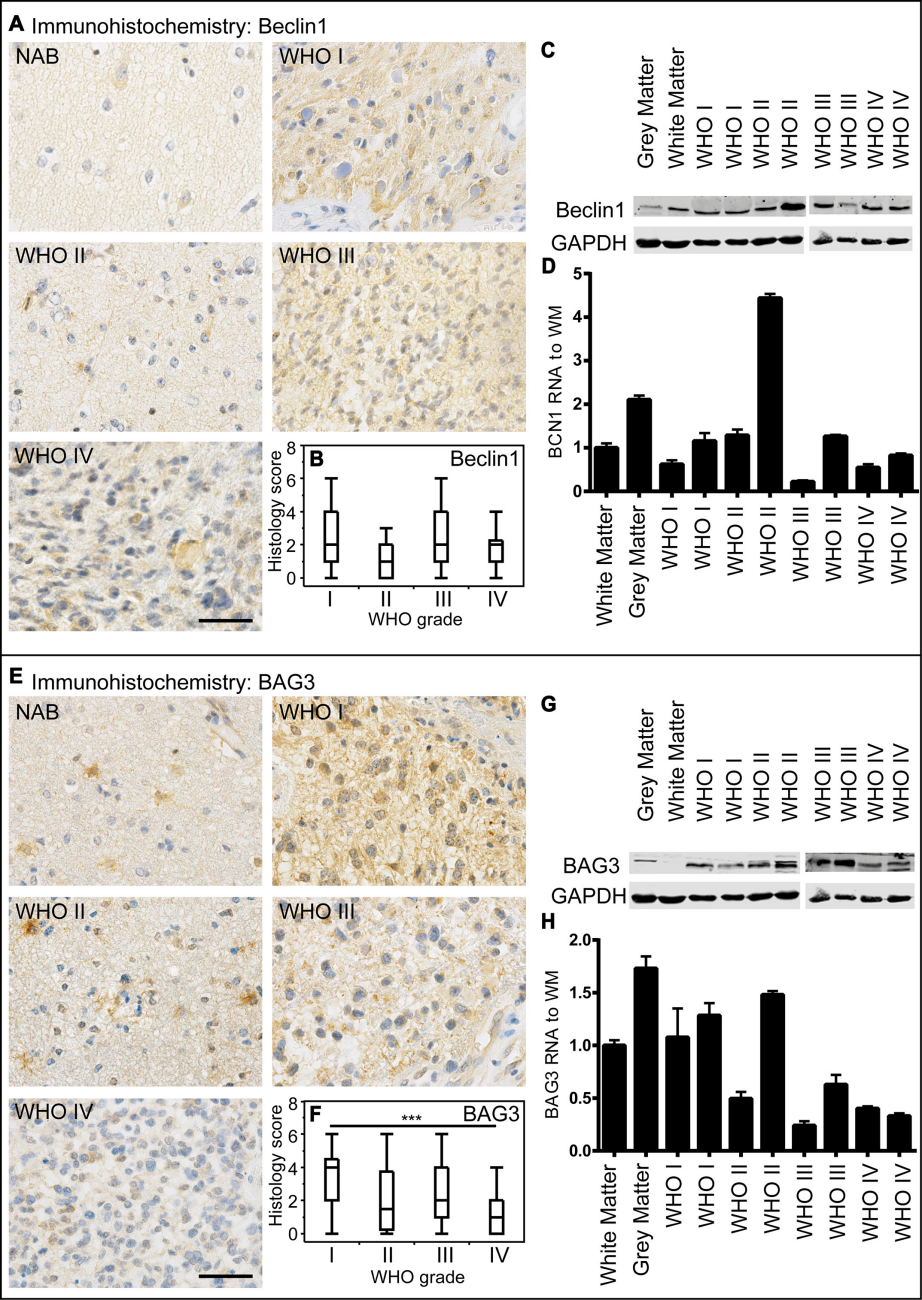
Beclin1 and BAG3 expression in human astrocytomas (**A**) Representative immunohistochemical staining against Beclin1 of NAB, pilocytic astrocytoma WHO grade I, diffuse astrocytoma WHO grade II, anaplastic astrocytoma WHO grade III and glioblastoma WHO grade IV. Scale bar: 50 μm. (**B**) Box blots showing Beclin1 scores (frequency × intensity) in 47 pilocytic astrocytomas WHO grade I (min: 0, max: 9, median: 2), 9 diffuse astrocytomas WHO grade II (min: 0, max: 6, median: 1), 34 anaplastic astrocytomas WHO grade III (min: 0, max: 9, median: 2) and 242 glioblastomas WHO grade IV (min: 0, max: 12, median: 2) (**C**) Beclin1 immunoblotting of normal grey and white matter and two astrocytomas of each WHO grade. (**D**) Relative Beclin1 mRNA levels of normal grey and white matter and two astrocytomas of each WHO grade normalized to normal white matter assessed by qPCR. (**E**) Representative immunohistochemical staining against BAG3 of NAB, pilocytic astrocytoma WHO grade I, diffuse astrocytoma WHO grade II, anaplastic astrocytoma WHO grade III and glioblastoma WHO grade IV. Scale bar: 50 μm. (**F**) Box blots showing BAG3 scores (frequency × intensity) in 46 pilocytic astrocytomas WHO grade I (min: 0, max: 12, median: 4), 16 diffuse astrocytomas WHO grade II (min: 0, max: 6, median: 1.5), 34 anaplastic astrocytomas WHO grade III (min: 0, max: 6, median: 2) and 242 glioblastomas WHO grade IV (min: 0, max: 6, median: 1) (**G**) BAG3 immunoblotting of normal grey and white matter and two astrocytomas of each WHO grade. (**H**) Relative BAG3 mRNA levels of normal and white matter and two astrocytomas of each WHO grade normalized to normal white matter assessed by qPCR.

**Figure 4:**
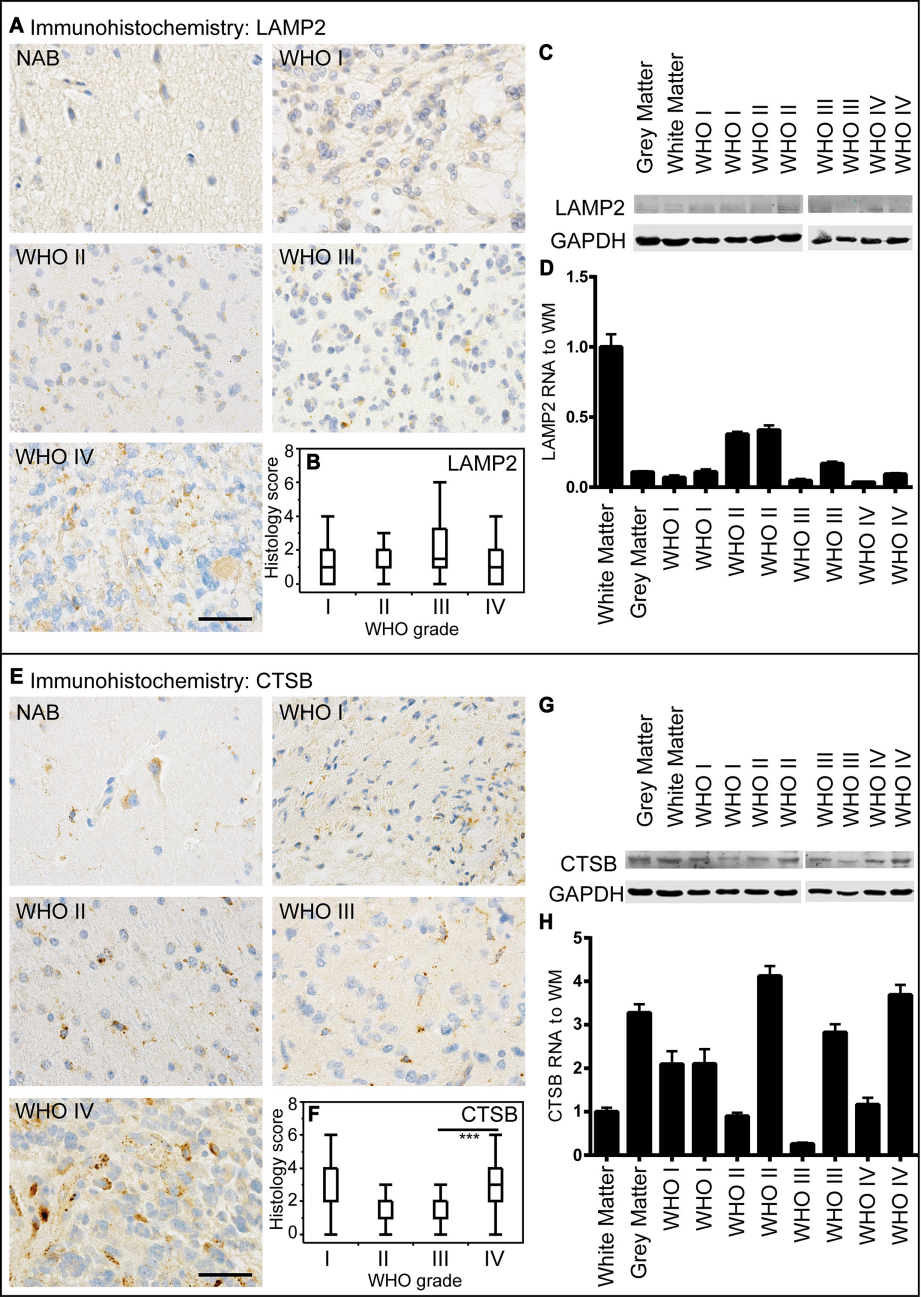
LAMP2 and CTSB expression in human astrocytomas (**A**) Representative immunohistochemical staining against LAMP2 of NAB, pilocytic astrocytoma WHO grade I, diffuse astrocytoma WHO grade II, anaplastic astrocytoma WHO grade III and glioblastoma WHO grade IV. Scale bar: 50 μm. (**B**) Box blots showing LAMP2 scores (frequency × intensity) in 45 pilocytic astrocytomas WHO grade I (min: 0, max: 12, median: 1), 16 diffuse astrocytomas WHO grade II (min: 0, max: 4, median: 2), 34 anaplastic astrocytomas WHO grade III (min: 0, max: 6, median: 1.5) and 247 glioblastomas WHO grade IV (min: 0, max: 12, median: 1). (**C**) LAMP2 immunoblotting of normal grey and white matter, and two astrocytomas of each WHO grade. (**D**) Relative LAMP2 mRNA levels of normal grey and white matter and two astrocytomas of each WHO grade normalized to normal white matter assessed by qPCR. (**E**) Representative immunohistochemical staining against Cathepsin B (CTSB) of NAB, pilocytic astrocytoma WHO grade I, diffuse astrocytoma WHO grade II, anaplastic astrocytoma WHO grade III and glioblastoma WHO grade IV. Scale bar: 50 μm. (**F**) Box blots showing CTSB scores (frequency × intensity) in 47 pilocytic astrocytomas WHO grade I (min: 0, max: 9, median: 2), 16 diffuse astrocytomas WHO grade II (min: 0, max: 6, median: 1), 34 anaplastic astrocytomas WHO grade III (min: 0, max: 4, median: 1) and 249 glioblastomas WHO grade IV (min: 0, max: 12, median: 3). (**G**) CTSB immunoblotting of normal grey and white matter and two astrocytomas of each WHO grade. (**H**) Relative CTSB mRNA levels of normal grey and white matter and two astrocytomas of each WHO grade normalized to normal white matter assessed by qPCR.

There is only poor data concerning the diagnostic and clinical relevance of the autophago-lysosomal network in human glioma tissue. To investigate the protein degradation machinery *in vivo*, we investigated normal appearing brain (NAB) tissue, glioblastoma infiltration zone and tumor tissue from astrocytoma patients and evaluated the protein expression of the key autophago-lysosomal factors LC3B, p62, Beclin1, BAG3, LAMP2 and CTSB. LC3B-positive punctae did not significantly differ amongst astrocytomas of different WHO grades (Figure 2A, 2B). These findings were confirmed by immunoblot and RT-PCR analyses (Figure 2C, 2D). In normal brain tissue LC3B protein expression was mainly restricted to neuronal cells while most glial cells remained negative (Figure 2A). Immunoblotting (Figure 2C) and mRNA analysis (Figure 2D) displayed slightly lower LC3B expression levels while the conversion of LC3B-I to LC3B-II was higher in glioma tissue as compared to normal brain (Figure 2C). We further compared LC3B expression regarding its location within the brain of glioblastoma patients including normal appearing brain tissue, infiltration zone and corresponding tumor centers (Supplementary Figure S7A). LC3B protein levels were significantly higher in the tumor center as compared to remote normal appearing brain tissue. Similarly to LC3B, p62 expression levels did not considerably vary between astrocytomas of different WHO grades (Figure 2E–2H) but were higher in glioblastoma centers as compared to remote normal appearing brain tissue (Supplementary Figure S7B). Beclin1 protein levels were slightly higher in human astrocytomas as compared to LC3B and p62, however did also not considerably differ amongst astrocytomas of different WHO grades (Figure 3A–3D). Furthermore, Beclin1 levels were significantly lower in normal appearing white matter as compared to corresponding grey matter and glioblastoma infiltration zones (Supplementary Figure S7C). In contrast to all other autophago-lysosomal factors, BAG3 showed significantly stronger staining levels in low-grade pilocytic astrocytoma (Figure 3E–3H) as well as normal appearing brain tissue and infiltration zone than in glioblastoma tumor centers (Supplementary Figure S7D). Morphologically, BAG3 protein levels most likely originated from reactive astrocytes, which showed distinct signals in immunohistochemical stainings (data not shown). Concerning the classic lysosomal markers LAMP2 (Figure 4A–4D and Supplementary Figure S7E) and CTSB (Figure 4E–4H and Supplementary Figure S7F) no considerable differences were observed in LAMP2 protein (Figure 4A–4C) and RNA expression (Figure 4D), while slightly higher CTSB protein levels were detected in glioblastoma tumor centers compared to normal CNS tissue and infiltration zones (Supplementary Figure S7F). WHO grade III astrocytomas also showed decreased CTSB levels as compared to WHO grade IV glioblastoma (Figure 4E). We next assessed the clinical relevance of autophago-lysosomal markers in the most malignant glioma variant, the glioblastoma (Supplementary Figure S8). We used the median split for protein expression of each factor to stratify our patient cohort into two subgroups. Patient survival curves only revealed an association of prolonged patient survival in case of high LAMP2 expression levels (Supplementary Figure S8E).

### LC3B, p62, LAMP2 and CTSB are loco-regionally upregulated in glioblastomas in oxygen- and nutrient-deprived areas

**Figure 5:**
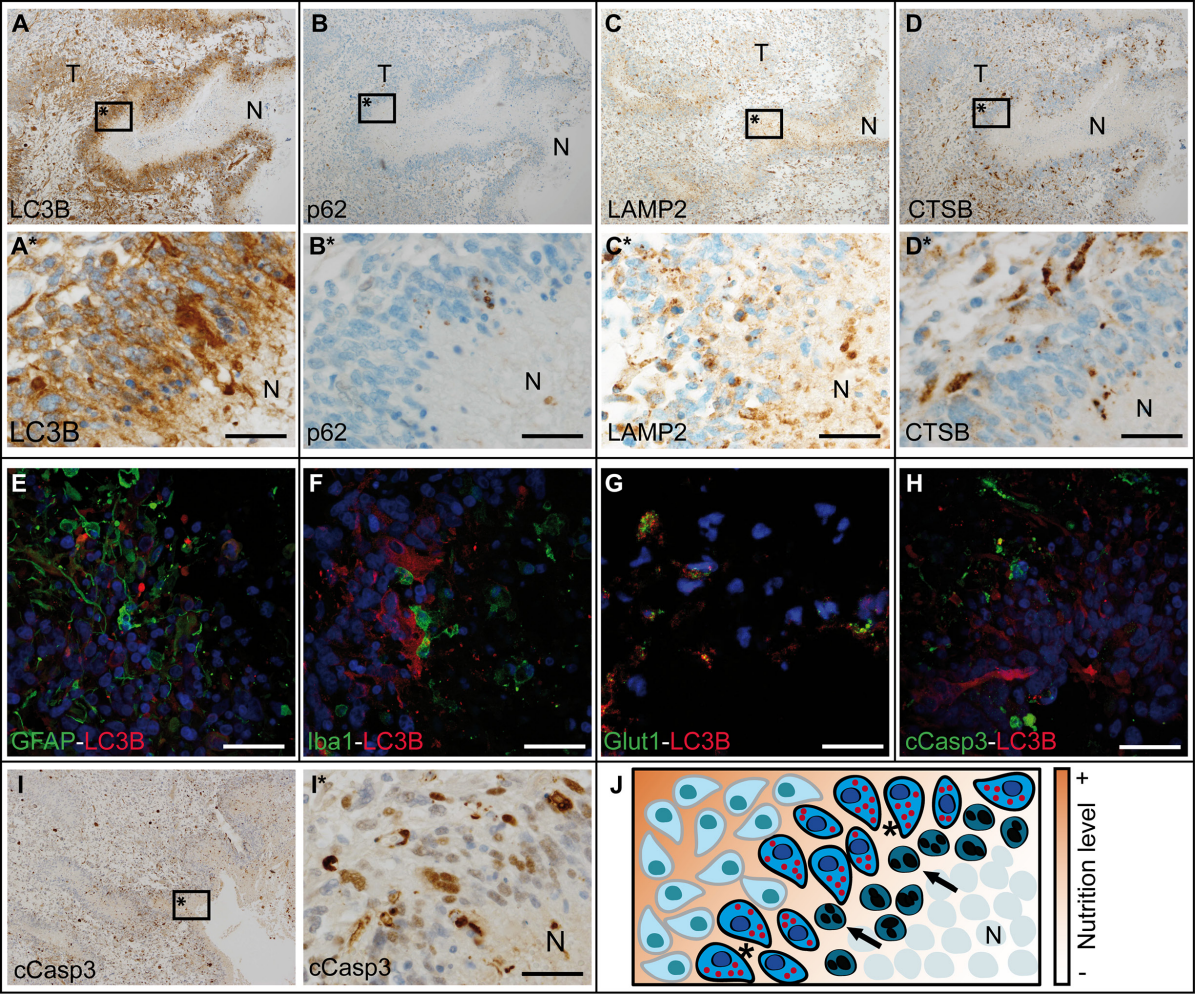
Autophago-lysosomal proteins are upregulated in close vicinity to necrotic foci in glioblastoma Overview about (**A**) LC3B, (**B**) p62, (**C**) LAMP2 and (**D**) CTSB immunohistochemistry in glioblastoma (N: necrosis, T: tumor center). (E–H) Double immunofluorescent staining against LC3B and (**E**) GFAP, (**F**) Iba1, (**G**) Glut1 as well as (**H**) cCasp3 in glioblastoma. (**I**) Overview of cCasp3 immunohistochemistry in glioblastoma. (A*, B*, C*, D* and I* are higher magnifications of A, B, C, D and I respectively; all scale bars: 50 μm). (**J**) Schematic overview of the border zone of necrotic foci with different nutrition levels in glioblastoma (arrows: apoptotic cell, *cells expressing autophagy-associated and lysosomal markers, N: necrosis).

Since, in general, no considerable differences were detected in ALP activation, we investigated tumor areas with prominent activation of the ALP in more detail. Highest LC3B (Figure 5A, 5A*), p62 (Figure 5B, 5B*), LAMP2 (Figure 5C, 5C*) and CTSB (Figure 5D, 5D*) levels were seen in close vicinity of pseudopalisading necrosis in human glioblastomas, frequently decreasing within a 100–500 μm distance from the border of the pseudopalisading cells. This could indicate a secondary, oxygen- and/or nutrient-dependent upregulation of these proteins rather than an *a priori* tumor phenotype. Double immunofluorescent stainings deciphered GFAP-positive glioma cells as major source of LC3B punctae formation next to necrotic foci (Figure 5E), whereas Iba1-positive microglia/glioma-associated macrophages were mainly devoid of LC3B expression (Figure 5F). To address the question if LC3B is associated with glioma cells suffering from hypoxia and glucose deprivation, we used the glucose transporter Glut1 as a reliable sensor for both conditions [19]. The strong co-localization of LC3B with Glut1 (Figure 5G) presumably indicates that the detection of LC3B in GBM *in vivo* is mainly related to a cellular state of hypoxia and malnutrition. Cells undergoing apoptosis as indicated by cleaved caspase 3 (cCasp3) staining did not overlap with cells that displayed strong LC3B punctae formation (Figure 5H). Similar co-localization results were obtained for the autophagic cargo receptor and adapter protein p62 (Supplementary Figure S9). Between the cell layers with prominent ALP activation and necrotic foci, prominent levels of cleaved caspase 3 (cCasp3), an indicator of apoptosis, were detectable (Figure 5I, 5I*). The distinct distribution pattern of activated ALP and apoptotic pathways related to hypoxia and malnutrition are schematically summarized in Figure 5J.

### Glucose depletion is a more potent inducer of ALP than hypoxia in glioma cells

**Figure 6:**
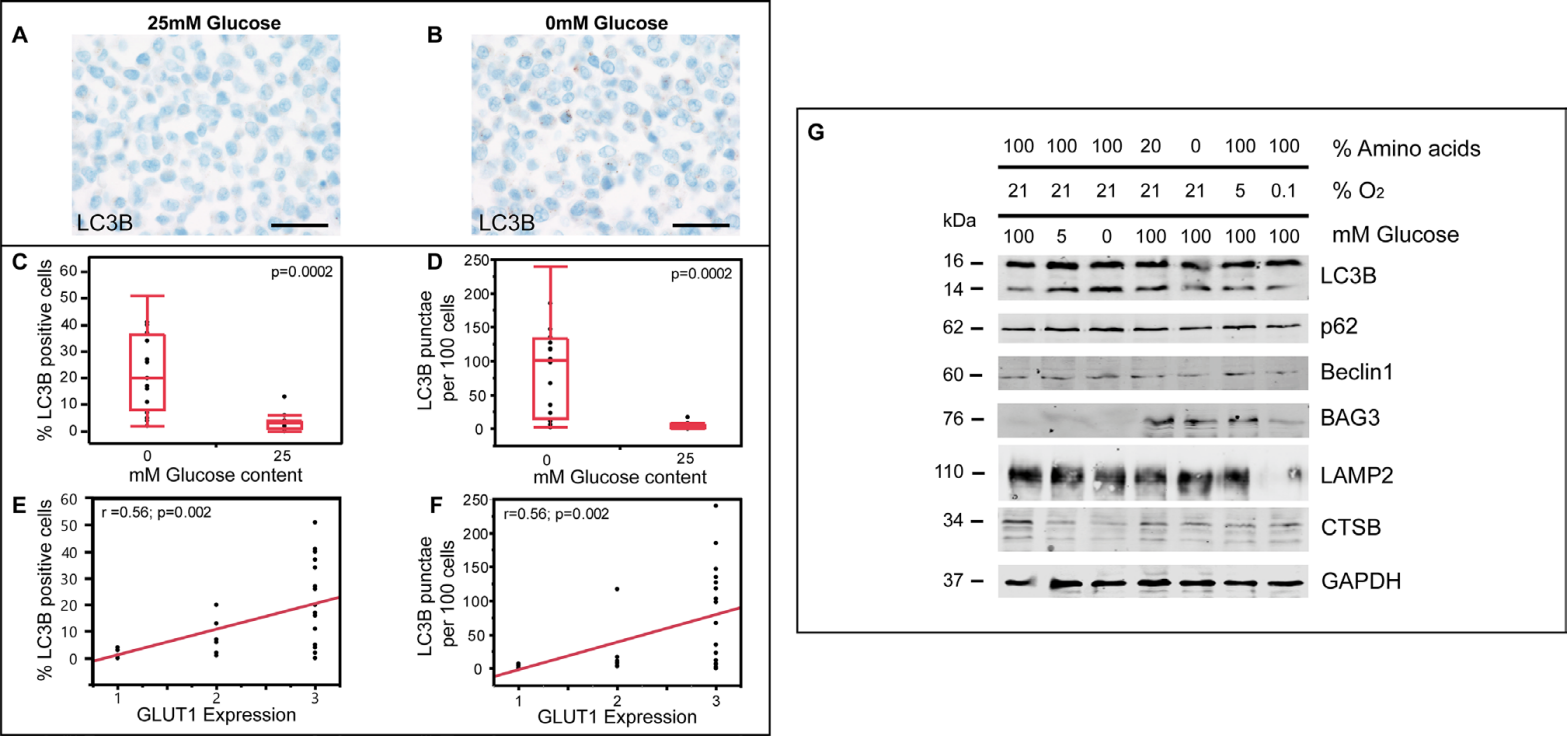
Glucose depletion is a more potent inducer of ALP than hypoxia in glioma cells (**A–B**) LC3B immunohistochemistry of LNT-229 cells cultured in serum-free medium (A) with 25 mM glucose and (B) without glucose (scale bar: 50 μm). (**C**) Percentage of LC3B positive cells in immunocytochemistry of cell culture conditions with 0 (min: 2, max: 51, median: 20) and 25 mM glucose (min: 0, max: 13, median: 3) from 28 differently treated LNT-229 cytopellets. (**D**) LC3B punctae per 100 cells in immunocytochemistry of cell culture conditions with 0 (min: 2, max: 240, median: 100.5) and 25 mM glucose (min: 0, max: 17, median: 4.5) from 28 differently treated LNT-229 cytopellets. Different treatment options: see Supplementery Table S1. (E, F) Correlation of Glut1 (1 = weak, 2 = moderate, 3 = strong) expression and (**E**) LC3B-positive cells or (**F**) LC3B-positive punctae per 100 cells in 28 differently treated LNT-229 cell culture experiments. (**G**) Immunoblotting of LNT-229 cells cultured in serum-free medium at different oxygen, glucose or amino acid (arginin, lysine, L-glutamine) levels.

To further mechanistically elucidate the major drivers for ALP induction in glioma cells, we used a cell culture-based system allowing for the modulation of oxygen and nutrient levels. While LNT-229 glioma cells were almost devoid of LC3B-positive punctae under 25 mM glucose, glucose starvation (0 mM glucose) induced a considerable amount of LC3B-positive punctae (Figure 6A, 6B). For quantification of these findings, we used a cytopellet micro array including varying glucose and oxygen levels (Supplementary Table S1). The quantification of LC3B-positive punctae in immunocytochemical stainings revealed that both the number of LC3B-positive cells (Figure 6C) as well as the number of LC3B-positive punctae per 100 cells (Figure 6D) were significantly increased upon glucose restriction and largely independent from additional treatment conditions. In addition, both number of LC3B-positive cells (Figure 6E) as well as the number of LC3B-positive punctae per 100 cells (Figure 6F) significantly correlated with Glut1 expression levels. We further separately assessed effects of glucose, oxygen and amino acid levels on ALP activation. Decreasing glucose levels considerably increased LC3B turnover as compared to oxygen or amino acid deprivation while Beclin1 and p62 levels remained almost unchanged (Figure 6G, Supplementary Figure S3A–S3H, Supplementary Figure S4A–S4D).

### Apoptosis and autophagy are independently induced in glioma cells

**Figure 7:**
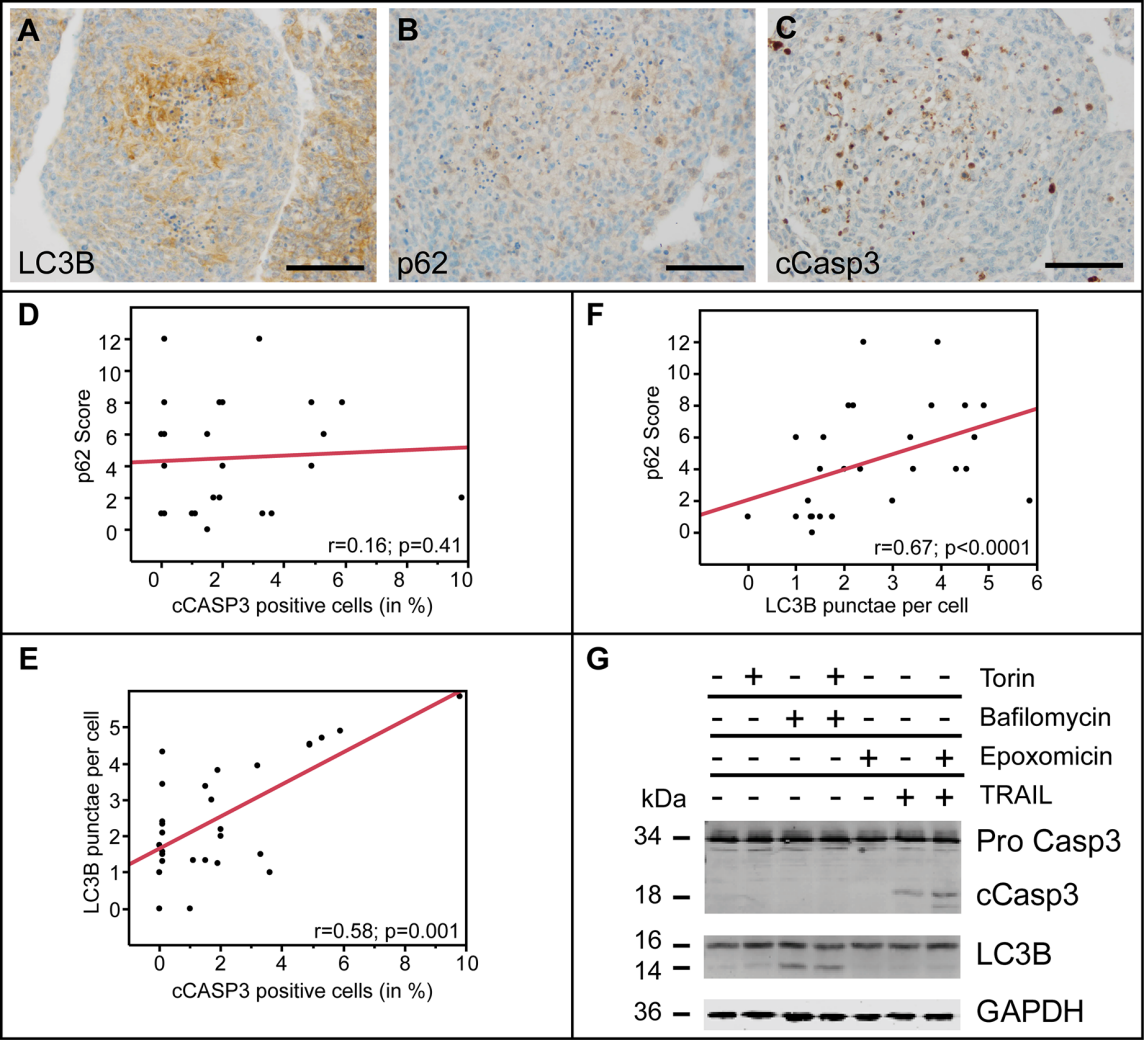
Autophago-lysosomal and apoptotic pathways are independently induced in glioma cells (**A**) LC3B, (**B**) p62 and (**C**) cCasp3 immunocytochemistry of primary glioblastoma spheres (scale bars: 100 μm). (**D**) Correlation cCasp3-positive cells (in %) and p62 Score (frequency × intensity) in 28 differently treated LNT-229 cell culture experiments. (**E**) Correlation analysis of cCasp3-positive cells (in %) and absolute numbers of LC3B-positive punctae per cell in 28 differently treated LNT-229 cell culture experiments. (**F**) Correlation analysis of LC3B punctae per cell and p62 score (frequency × intensity) in 28 differently treated LNT-229 cell culture experiments. (**G**) Immunoblotting of LNT-229 cells treated with Torin2 (250 nM, 8 h), BafA1 (100 nM, 8 h), Epoxomicin (50 nM, 18 h) or TRAIL (250 ng/ml, 18 h). DMSO served as a control.

There is an ongoing discussion about whether autophagy is induced in glioblastoma cells to promote survival and escape malnutrition or if it might also exert primarily alternative cell-death (type-II cell death) functions. By producing large tumor spheres from primary glioblastoma cells, we mimicked malnutrition and decreasing oxygen levels found in *in-vivo* in gliomas. The highest frequency of LC3B-positive punctae was detected in the center of primary glioma spheres reaching a diameter of up to 500 μm, whereas the sphere borders with direct contact to cell culture medium were almost completely devoid of LC3B (Figure 7A). The weakest p62 expression was also seen at the sphere surface, however central p62-positivity showed a slightly inverse gradient as compared to LC3B with lower expression levels in the most inner areas (Figure 7B). In contrast, the distribution of cCasp3-positive cells was more heterogeneous, with a trend towards the center of glioma spheres. In our cytopellet micro array analysis LC3B punctae formation, but not p62 expression positively correlated with cCasp3 (Figure 7D, 7E), while LC3B and p62 correlated positively (Figure 7F). This suggests that apoptotic cell death and autophagy induction in glioma cells are largely independent from each other. To further analyze in more detail if proteins of the ALP are activated simultaneously or independently from (apoptotic) cell death induction in general, we induced autophagy in LNT-229 glioma cells to observe whether apoptosis is activated or vice versa (Figure 7G). Induction of autophagy by Torin2 showed the expected LC3B-I to -II conversion but no caspase 3 cleavage as an indicator of apoptosis in immunoblotting analysis. Treating LNT-229 with TRAIL, a specific apoptosis inductor led to increased caspase cleavage - as expected - but not to LC3B-I to -II conversion. The proteasome inhibitor Epoxomicin served as enhancer of TRAIL (Figure 7G). These results corroborate the independent regulation of autophagy and apoptosis in glioma cells.

## DISCUSSION

Studying autophago-lysosomal pathway (ALP) activation is a hot topic in current cancer research and there is increasing evidence that glioma cells might react to anti-cancer treatment rather via induction of ALP than by apoptosis [20]. In recent years there has been a lot of debate whether inhibition [21] or stimulation [22] of ALP might be a useful anti-glioma treatment strategy [23]. It has been shown that inhibition of autophagy at early steps reduced the effects of chemotherapeutic treatment with TMZ while late-stage inhibition rendered glioma cells more susceptible [24]. To date, fast, reliable and standardized methods to study ALP induction in the daily (neuro-) pathological diagnostic setting are missing. There is a strong need for diagnostic tools reflecting ALP induction at light microscopic level since rather time-consuming electron microscopy only provides a very restricted regional overview and LC3B-I to -II conversion in immunoblot analyses do not allow to detect cell-type specific differences [20]. In our study we established a reliable immunohistochemical diagnostic platform corroborated by qPCR, immunoblotting, and immunofluorescence to assess autophagy-associated and lysosomal markers (Figure 1, Supplementary Figures S1 and S6). Our panel comprised antibodies against different steps of the ALP and were directed against (i) a very early inducer of autophagy: Beclin1 [25], (ii) the selective autophagy receptor for ubiquitinated proteins: p62/SQSTM1 [26], (iii) the key molecule for the elongation of autophagosomal membranes: LC3B [27], (iv) a central non-canonical stimulator of selective autophagy: BAG3 [28] as well as (v) Cathepsin B and (vi) LAMP2 both necessary for a well-functioning autophagic flux and lysosome homeostasis [29]. Although being present only at very low levels in human gliomas in general, both key factors of the initial steps of ALP activation, namely p62 and LC3B, were more prominently expressed in WHO grade I to IV gliomas at protein level detected with immunohistochemistry as compared to normal CNS specimens (Supplementary Figure S7A, S7B). Additionally, no significant differences were found between the grades of malignancy. These findings are in contrast to *in vitro* studies presenting p62 as a major player for the migratory/invasive behavior of so-called glioblastoma initiating cells [30]. In our large cohort of human glioma samples, also the non-migratory pilocytic astrocytoma samples displayed similar p62 levels as compared to their diffusely infiltrating counterparts of WHO grades II–IV both in immunocytochemical and biochemical analyses. It has been previously reported in a small cohort of glioblastoma patients that only about a quarter of them displayed extensive LC3B expression without providing further details about the intra-tumoral variations [31]. In our study, ALP activation seems to be very low in general and strong induction is associated with nutrient-deprived areas (Figure 5) rather than any stem or progenitor cell phenotype within the tumors. Furthermore, it has not yet been clarified to which extent ALP activation seen in biochemical analyses derives from glioma cells or from cells of the glioma microenvironment such as glioma-associated macrophages/microglia [32]. In line with those findings, our results also display glioma cells (Figure 5E) as major source of early ALP activation in human gliomas while glioma-associated cells of the myeloid lineage were mainly devoid of prominent ALP signals (Figure 5F). In contrast to the findings for p62 and LC3B that stronger ALP activation was seen in glioma tissues as compared to normal brain, no differences were found for Beclin1 which is known to be a very early inducer of autophagy (Figure 3A–3D and Supplementary Figure S7). These findings might at least be partly explained by very recent data showing that Beclin1 is not a prerequisite for LC3B lipidation however its absence might lead to malformation of autophagosomes [33]. In an experimental study based on alkylating agents, it has been demonstrated that the degradation of Beclin1 had no effect on the level of autophagy pointing to potential alternative ALP activation mechanisms in gliomas [34]. For BAG3, a non-canonical inducer of autophagy, even higher expression levels were found in normal appearing glioma-adjacent tissues and lowest grade pilocytic astrocytomas, WHO grade I (Figure 3E–3H and Supplementary Figure S7D). Morphologically, BAG3 was mainly restricted to reactive astrocytes. These findings are in line with previous studies that deciphered reactive astrocytes as source of BAG3 expression in CNS ischemia models [35]. Taken together, BAG3 seems to contribute only to a very limited extent to ALP activation in the CNS under normal and neoplastic conditions. The key factors which are necessary for completion of the autophagic flux and proper lysosomal functioning, CTSB and LAMP2, were also expressed to an almost similar extent as p62 and LC3B (Figure 4 and Supplementary Figure S7E, S7F). The intra-tumoral distribution of CTSB and LAMP2 nicely paralleled the heterogeneity of p62 and LC3B indicating that central constituents of the ALN are present in similar areas in human gliomas thereby allowing for the functioning of the entire autophago-lysosomal degradation complex (Figure 5). As ALP activation roughly correlated with areas of prominent apoptosis induction in human gliomas (Figure 5I, 5J), we aimed at further deciphering (i) if apoptosis and autophagy are induced simultaneously in glioma cells and (ii) the major underlying drivers for ALP induction. The first question is of importance since the induction of autophagic vacuoles has been demonstrated to precede apoptotic cell death pointing to common pathways in the induction of autophagy and apoptosis [36]. Although areas showing apoptosis or autophagy roughly correlated *in vivo*, no overlap of ALP activation and apoptosis could be detected at a cellular level using a glioma sphere model mimicking areas of enough and impaired oxygen and nutrient supply (Figure 7). LC3B induction considerably increased with the distance from the sphere surface indicating a continuous accumulation with decreasing nutrient supply. Apoptotic cells were more randomly distributed within glioma spheres with increasing density of cCasp3-positive cells in the inner layer of the glioma spheres (Figure 7C). The strongest p62 expression was found in the area surrounding the strongly LC3B-positive sphere center (Figure 7A, 7B). We further identified restricted nutrient supply as major factor for ALP activation whereas severe hypoxia (in the presence of excessive glucose) or amino acid restriction showed almost no effect on ALP and even a slight decrease of LC3B and p62 (Figure 6). That is in line with previous data demonstrating that hypoxia-activated autophagy may even enhance the degradation of p62 [37]. In our experiments, glucose restriction was a major activator of autophagy supporting the concept that tumor cells very strongly rely on glucose metabolism, the so-called Warburg hypothesis [38]. This theory is further corroborated by our findings that the expression of the glucose transporter Glut1 significantly positively correlated with the amount of LC3B-positive punctae in glioma cells (Figure 6E, 6F). In addition, the p53 mutation status which has been shown to be an important upstream regulator of the ALN and also a driver in glioma development [5, 6] did not have a considerable impact on ALP induction in glioma cells neither upon glucose restriction nor amino acid starvation (Supplementary Figure S2–S5). Our data show that ALP activation is most likely a secondary process induced by micro-environmental conditions in human gliomas and no considerable association with patient prognosis or grade of malignancy was observed. This is in contrast to data from *in vitro* experiments or other cancer entities such as lymphoma or melanoma [39].

In summary, our study provides a reliable platform for the assessment of ALP activation *in vivo* in human tissue specimens at the cellular level. The central ALP axis including p62-LC3B-CTSB-LAMP2 was generally activated in a similar manner while Beclin1 levels, an alternative inducer of autophagy, did not considerbly differ upon ALP induction in human gliomas. Furthermore, the non-canonical ALP stimulator BAG3 was mainly restricted to areas showing reactive gliosis in both normal appearing brain tissue as well as glioma tissues. We could also demonstrate that ALP activation locates to peri-necrotic, glucose-restricted areas in GBM. When taking the whole tumor section into account, no considerable association with patient outcome or grade of malignancy was detectable. In conclusion, our data may at least partly explain why first clinical phase I/II studies in glioblastomas showed rather disappointing results [15] with regard to both the specific success of autophagy inhibition and in general the impact on patient survival. Our findings further highlight a strong impact of the glioma microenvironment on the extent of ALP activation in these tumors.

## MATERIALS AND METHODS

### Patient characteristics and tissue specimens

We investigated 350 brain tumor samples collected at the University Hospital Frankfurt am Main, Germany. Our cohort comprised pilocytic astrocytomas WHO grade I (*n* = 47), diffuse astrocytomas WHO grade II (*n* = 16), anaplastic astrocytomas WHO grade III (*n* = 35) and glioblastomas WHO grade IV (*n* = 252) (for summary see Table 1). Normal appearing CNS tissue specimens remote to the tumor core and autopsy cases without clinical or neuropathological evidence for CNS pathologies were used as controls. The use of patient material was approved by the ethical committee of the Goethe University Frankfurt, Germany (GS 04/09 and SNO-06-2014). Neuropathological diagnostics was performed by at least 2 experienced neuropathologists (KHP, PNH, MM) according to the current WHO classification for tumors of the central nervous system [40]. Immunohistochemical analyses of patients material was performed on tissue micro arrays as previously published [41].

**Table 1:** Summary of tissue specimens and patient data

	Pilocytic astrocytoma WHO°I	Diffuse astrocytoma WHO°II	Anaplastic astrocytoma WHO°III	Glioblastoma WHO°IV	Infiltration zone of glioblastoma	Surrounding normal appearing white matter	Surrounding normal appearing grey matter
**Male/female**	17/30	10/6	20/15	143/109	23/16	9/10	32/30
**Median age (range)**	14.0 (0–75)	30.5 (5–52)	43 (22–66)	61 (7–80)	61 (8–79)	63 (8–78)	61 (30–78)
**Specimens (*n*)**	47	16	35	252	39	19	62
**Tumor localisation (supratentorial/infratentorial)**	31/16	16/0	34/1	250/2	39/0	19/0	60/0
**median follow-up (range) in months**	31.0 (0.0–142.1)	52.9 (0.0–122.1)	12.1 (0.0–164.3)	11.3 (0.0–94.0)	5.9 (0.0–47.8)	6.8 (0.0–34.6)	9.1 (0.0–104.7)

Abbreviation: CI: confidence interval.

### Cell culture and reagents

As the human malignant glioma cell line LN-229 is p53 wild-type which is in contrast to was has been previously published, the cells used in our experiments were renamed LNT-229 for clarification (T stands for Tuebingen) [42]. In addition, we used cell lines (MZ-18, T98G and U87) with different p53 status to show a potential association of this mutation with ALN factors. Cells were cultured in 75 cm² cellstar plastic flasks using Dulbeccos's modified Eagle's medium (DMEM) supplemented with 25 mM Glucose, 1% glutamine (Gibco DMEM Glutamax, Invitrogen, Carlsbad, CA, USA), 10% FCS (Biochrom, Berlin, Germany), penicillin/streptomycin (each 100 μg/ml, Sigma, Deisenhofen, Germany) and 1 mM pyruvate (Sigma). Cell culture supplements such as glutamine or pyruvate content were adapted to the appropriate experiment.

### Cytopellets

Cytopellets were generated by centrifuging the cells or spheres after treatment at 350 g for 3 min. Supernatants were discarded and pellets fixed in 4% buffered formalin (pH 7.4) for 48 hours and afterwards embedded in paraffin.

### Primary glioma cells and tumor spheres

Human glioblastoma tissue obtained intraoperatively was immediately processed to generate primary glioma cells. DMEM-F12 medium contained 20 ng/ml of each recombinant epidermal growth factor (EGF) and basic fibroblast growth factor 2 (bFGF2) (Reliatech, Wolfenbüttel, Germany) as well as 20% BIT admixture 100 supplement (Pelo Biotech, Planegg/Martinsried, Germany). For cell cultivation, we used laminin-coated flasks (5 mg/ml, 3 h, Sigma).

### Immunocytochemistry and immunohistochemistry

Specimens were fixed in 4% buffered formalin (pH 7.4) and embedded in paraffin (Tissue-Tek VIP, Sakura, Alphen on the Rhine, Netherlands). For immunohistochemistry, an automated system with established standard protocols of our group was used (Ventana DiscoveryXT, Roche, Strasbourg, France). The immuno-staining protocols have been previously described in detail [41]. The following primary antibodies were used: anti-human LC3B (mouse antibody, 0260–100, dilution: 1:100, NanoTools, Teningen, Germany), anti-human p62 (mouse antibody, 610832, dilution: 1:500, BD Transduction Laboratories, Franklin Lakes, NJ, USA), anti-human BAG3 (rabbit antibody, PAB0330 dilution: 1:100, Abnova, Taipei City, Taiwan), anti-human Beclin1 (rabbit antibody, 0260–100, dilution: 1:100, Santa Cruz, Dallas, TX, USA), anti-human LAMP2 (rabbit antibody, ab37024, dilution: 1:200, Abcam, Cambridge, MA, USA), anti-human Cathepsin B (goat antibody, sc-6493, dilution: 1:250, Santa Cruz) and anti-human cleaved caspase 3 (rabbit antibody, dilution: 1:100, Cell Signaling, Cambridge, UK). As an isotype control for monoclonal mouse antibodies, anti-Mouse-IgG1 (X0931, Dako, Glostrup, Denmark) was used at the same concentration as the specific primary antibody.

### Light microscopy and scoring

In order to assess the immunohistochemical stainings, a semi-quantitative score was applied taking into account both staining frequency and intensity. The staining frequency scores 0 (0–1%), 1 (1–10%), 2 (10–25%), 3 (25–50%) and 4 (> 50%) were multiplied with the intensity scores, the latter ranging from weak (1) over moderate (2) to strong (3) as previously described [43]. Tissue sections and cytopellets were analysed using an Olympus BX50 light microscope (Olympus, Hamburg, Germany).

### Immunofluorescence

Slides were deparaffinized and dehydrated with Xylol (Roth, Karlsruhe, Germany) and alcoholic diluents before cooking them in citrate buffer for 40 min. Afterwards, the compounds were blocked with Roti^®^-Block (Roth) and both primary antibodies (for LC3B, p62 see immunocytochemistry and immunohistochemistry). Anti-human Iba1 (rabbit antibody, 019-10741, dilution: 1:1000, Wako, Osaka, Japan), anti-human Glut1 (rabbit antibody, ab 652, dilution: 1:100, Abcam, Cambridge, UK), anti-GFAP (rabbit antibody, Z0334, dilution: 1:1000, Dako, Glostrup, Denmark) were applied for 1 h. First and second secondary antibodies (Alexa Fluor goat anti-mouse 568, Alexa Fluor goat anti-rabbit 488, dilution 1:500, Invitrogen, Carlsbad, CA, USA) were applied separately for 1 h, each. Cell nuclei were stained with Topro 3 (dilution: 1:1000, Invitrogen) and DAPI (4′,6-diamidino-2-phenylindole, dilution: 1:500, D1306, Invitrogen) thereby enabling to use both confocal as well as fluorescence microscopy. Finally, slides were mounted with cover slips (24 × 36 mm or 24 × 50 mm; depending on compound size; Knittel, Bielefeld, Germany) using Aqua-Poly/Mount (Fluorescent Mounting Medium, Dako). Slides were rinsed in PBS three times for 5 min between each working step to prevent drying out as well as reagent contamination or mixture. Fluorescence images were analyzed and recorded using a Nikon C1si (Nikon, Tokyo Japan) confocal microscope and NIS elements software. After recording, digital images were processed and adjusted for brightness, contrast and color balance.

### Opera immunofluorescence analysis

LNT-229 cells were seeded on 384 Well Plates (Perkin Elmer, Waltham, MA, USA) and fixed with 4% paraformaldehyde. Cells were permeabilized with 0.5% Triton-X 100 (VWR, Darmstadt, Germany) in PBS (10 min) followed by blocking with 1% BSA in PBS (Gibco) for 1 h. Primary (anti-LAMP2, ab25631, Abcam); anti-LC3B (Cell Signaling #2775); anti-p62 (mouse antibody, M162-3, MBL International, Woburn, MA, USA)) and secondary antibodies as well as nuclear and cytoplasmic staining reagents (Alexa Fluor-coupled antibodies (Life Technologies); DRAQ5 (Cell Signaling); HSC Cell Mask Deep red stain (Life Technologies)) were incubated in 0.1% BSA in PBS for 1 h with three washes of PBS in between. Images were acquired on PerkinElmer's Opera High Content Screening System with a 60× water-immersion objective and visualized with Acapella High Content Imaging Analysis software (Perkin Elmer). The number of antibody-labeled spots per cell were automatically counted using Acapella software.

### Immunoblotting

Fresh frozen patient specimens used for immunoblotting were evaluated for adequate tumor or normal brain tissue by two neuropathologists (PNH, MM) before preparing the tissue lysate. We aimed at reaching a vital tumor cell mass of > 70% as well as none or only minimal amount of necrosis. Lysates were produced from cell lines using RIPA buffer (50 mM, Tris pH 7.5, 150 mM NaCl, 0.1% SDS, 0.5% sodium desoxycholate, 1% NP40) with protease- and phosphatase inhibitors (HALT, ThermoScientific, Waltham, MA, USA), and used for immunoblotting. Cell cultures were controlled for cell density before treatment, to assure comparability. Protein concentration was determined using the Bradford assay (Thermo Scientific). For immunoblotting of patient specimens the following primary antibodies were used: anti-human LC3B (rabbit antibody, L8918, Sigma-Aldrich, St Louis, MO, USA), anti-human p62 (mouse antibody, 610832, BD Transduction Laboratories), anti-human GAPDH (mouse antibody, CB1001, Calbiochem, Billerica, MA, USA), anti-human BAG3 (rabbit antibody, PAB0330, Biozol/Abnova, Eching, Germany), anti-human Beclin1 (mouse antibody, m612112, BD Pharmingen, Franklin Lakes, NJ, USA), anti-human CathepsinB (goat antibody, sc-6493, Santa Cruz), anti-human LAMP2 (mouse antibody, ab25631, Abcam). To analyze the cell culture derived lysates, the following antibodies were used: anti-human LC3B (mouse antibody, #2775, Cell Signaling), anti-human LC3B (rabbit antibody, PM036, MBL International), anti-human p62 (mouse antibody, M162-3, MBL International), anti-human PCNA (rabbit antibody, sc-7907, Santa Cruz), anti-human cleaved caspase 3 (rabbit antibody, #9665 Cell Signaling), anti-human CathepsinB (goat antibody, sc-6493, Santa Cruz), anti-human Actin (mouse antibody, A4700 Sigma Aldrich), anti-human LC3B (Figure 6–7, rabbit antibody, L8918, Sigma Aldrich), anti-human p62 (Figure 6, mouse antibody, 610832, BD Transduction Laboratories), anti-human BAG3 (rabbit antibody, PAB0330, Biozol/Abnova), anti-human Beclin1 (mouse antibody, m612112, BD Pharmingen), anti-human LAMP2 (mouse antibody, ab25631, Abcam). Proteins were separated at 135 V by SDS-PAGE (self-casted 10%, 12% or 15% gels) and transferred to nitrocellulose membranes (NitroBind 0.45 μm, Fisher Scientific or Protean BA 83; 2 lm; Schleicher & Schuell, Dassel, Germany). Membranes were blocked with TBS-T (20 mM Tris; 150 mM NaCl; 0.1% Tween-20) containing 5% low fat milk (Roth). Blots were incubated with primary antibodies in blocking buffer at 4°C overnight and secondary HRP antibodies (anti-mouse-HRP antibody, W4021, Promega, Madison, USA, anti-rabbit-HRP antibody, W4011, Promega) or luminescent antibodies (Li-Cor: IRDye 680RD goat anti-rabbit and anti-mouse, IRDye 800 CW goat anti-rabbit and anti-mouse, Li-Cor, Bad Homburg, Germany) were added for 1 h after washing with TBS-T. Signal was detected with an Odyssey Imaging System (Li-Cor) or using enhanced chemiluminescence.

### RNA preparation and quantitative RT-PCR

Total RNA was extracted from cryomaterial obtained from glioma patients (normal appearing white matter, normal appearing grey matter, 2 samples from each WHO grade astrocytoma). We used peqGOLD TriFast reagent (peqlab, Erlangen, Germany) for RNA extraction. 1 μg RNA of each sample was then transcribed into cDNA using Fermentas cDNA synthesis kit (ThermoScientific, Waltham, MA, USA). The expression of the gene interest was assessed using SYBR green master mix (ThermoScientific), on a MyiQ Single Color Real-Time PCR Detection System (BIO-RAD, Hercules, CA, USA). Relative mRNAs expression were quantified as ∂∂CTs: ([E∂CT(gene)/E∂CT(RPLP0)]). For normalization, RPLP0 primers were used (forward: gagtcctggccttgtctgtgg; reverse: tccgactcttccttggcttca). White matter samples were used for calibration. For detailed information about all primers see Table 2.

**Table 2:** qPCR primers

RefSeq	Gene (strand)	Primer sequence	Product size (bp)
NM_003900.4	SQSTM1_01.s	TGAGGAAGATCGCCTTGGAGT	173
	SQSTM1_01.as	GTCCAGAGAGCTTGGCCCTTC	
NM_022818.4	MAP1LC3B_01.s	ATTCGAGAGCAGCATCCAACC	191
	MAP1LC3B_01.as	TGTCCGTTCACCAACAGGAAG	
NM_002294.2	LAMP2_01.s	ACACAACATTTCCTGATGCTGA	187
	LAMP2_01.as	TTTGTGCTCACTGTGCCATTT	
NM_001908.3	CTSB_01.s	GAGCAGGCCCTCTTTCCATC	188
	CTSB_01.as	GCAGCTTCAGGTCCTCGGTAA	
NM_004281.3	BAG3_02.s	CTCACCAGCCAGGAGCAGCA	195
	BAG3_02.as	CGGATCACTTGAATTGGGATGT	
NM_003766.3	BECN1_02.s	GCTGAGGGATGGAAGGGTCTAA	164
	BECN1_02.as	CGCCTGGGCTGTGGTAAGTAA	

Abbreviations: bp: base pairs; .s: sense strand; .as: antisense strand.

### Starvation and hypoxia cell culture experiments

For glucose deprivation and hypoxia experiments, cells were equally seeded in 75 cm² cellstar filter top plastic flasks and kept in the incubator overnight to ensure adherent growth. Expansion and treatment of the cells for starvation experiments was performed in DMEM medium (DMEM Life Technologies, Paisley, UK) containing 1 mM pyruvate (Sigma). For glucose deprivation experiments glucose- and serum-free medium (DMEM Life Technologies) was added to equally subconfluent cells. In the case of low glucose concentration, 5 mM of sterile D-(+)-glucose solution (45%, Sigma Aldrich, St. Louis, USA) was applied. For hypoxia experiments, cells were kept in serum-free medium and transferred into a hypoxia chamber (Binder incubator, Tuttlingen, Germany). The required oxygen concentration (0.1%, 5% and 21% O_2_) was set before the particular experimental condition. For amino acid starvation, DMEM medium lacking L-arginine, L-glutamine and L-lysine (DMEM Life Technologies) supplemented with 1 mM pyruvate (Sigma) was used. To generate the 20% amino acid condition, the appropriate amino acids (L-arginine hydrochloride and L-lysine hydrochloride from Sigma-Aldrich, St Louis, MO, USA and L-glutamine from Gibco, Invitrogen, Carlsbad, CA, USA) were added in 0.2 fold concentration of the control condition.

### Pharmacologic regulation of autophagy and apoptosis

To induce autophagy, the mTOR inhibitor Torin1 (250 nM, 2 h, Tocris, Bristol, UK) was used. Blocking of the autophagic flux was performed by using Bafilomycin A1 (100 nM, Sigma-Aldrich, St. Louis, USA). TRAIL (250 ng/ml for 18 h, Peprotech, Rocky Hill, NJ, USA) was used to induce apoptosis in combination with the proteasomal inhibitor Epoxomicin (50 nM, 18 h, Enzo, Lörrach, Germany) [44, 45]. DMSO (Sigma-Aldrich) served as negative control.

### Cytopellet micro array

To investigate autophagy further *in vivo* immunocytochemically, we established a cytopellet micro array by treating LNT-229 cells in different conditions with varying degrees of glucose, oxygen and glutamine levels, using a Hif1α knockdown, as well as by Torin2 (100 nM, 24 h or 48 h, Tocris) and Rapamycin (100 nM, 24 h or 48 h, Tocris) treatment. For Hif1α knockdown, Block-iT^™^ Lentiviral Pol II miR RNAi Expression Vector Kit system (K#4938-00, Invitrogen) was used. Control (KTR) cells were generated using pLenti6/V5-DEST plasmid (Invitrogen) targeting SIMA, the Drosophila homolog. Stable polyclonal cell lines were generated with 4 μg/ml Blasticidine (InvivoGen). For detailed list of condition combinations see Supplementary Table S1.

### Statistics

The semi-quantitative scores were assigned as an ordinal scale response variable and statistical differences among WHO grades, infiltration zones or normal appearing brain samples were assessed using the non-parametric Wilcoxon test for multiple comparisons. Survival analyses were performed using Kaplan-Meier analyses. In order to compare the survival curves we used Wilcoxon test for censored data. For correlation analyses we performed a linear fit, in case of ordinal scaled variables we used Spearman's rho (R) correlation analysis. A significance level of α = 0.05 was chosen for all tests. Statistical analysis was performed using JMP 11.0 software (SAS, Cary, NC, USA).

## SUPPLEMENTARY MATERIALS FIGURES AND TABLE


