# Supplementary material for: Diagnostic and clinical relevance of the autophago-lysosomal network in human gliomas
Source: Oncotarget. 2016 Mar 4;7(15):20016–32. doi: 10.18632/oncotarget.7910 (PMC4991435; doi:10.18632/oncotarget.7910)
Supplement: Supplementary file 1 [file oncotarget-07-20016-s001.pdf]

Diagnostic and clinical relevance of the autophago-lysosomal network in human gliomas

Supplementary Materials

Inducible 293TREx HEK cell culture

Inducible 293TRex HEK cells were generated using a lentiviral transduction system. Selection was determined with 4 µg/ml Blasticidine (InvivoGen, San Diego, CA, USA) and 1 µg/ml Puromycine (Sigma) added to DMEM supplemented with 10% fetal bovine serum, 0.2 mM L-glutamine and standard antibiotics. Induction was performed with 4 µg/ml doxycycline (Sigma) for 24 hours.

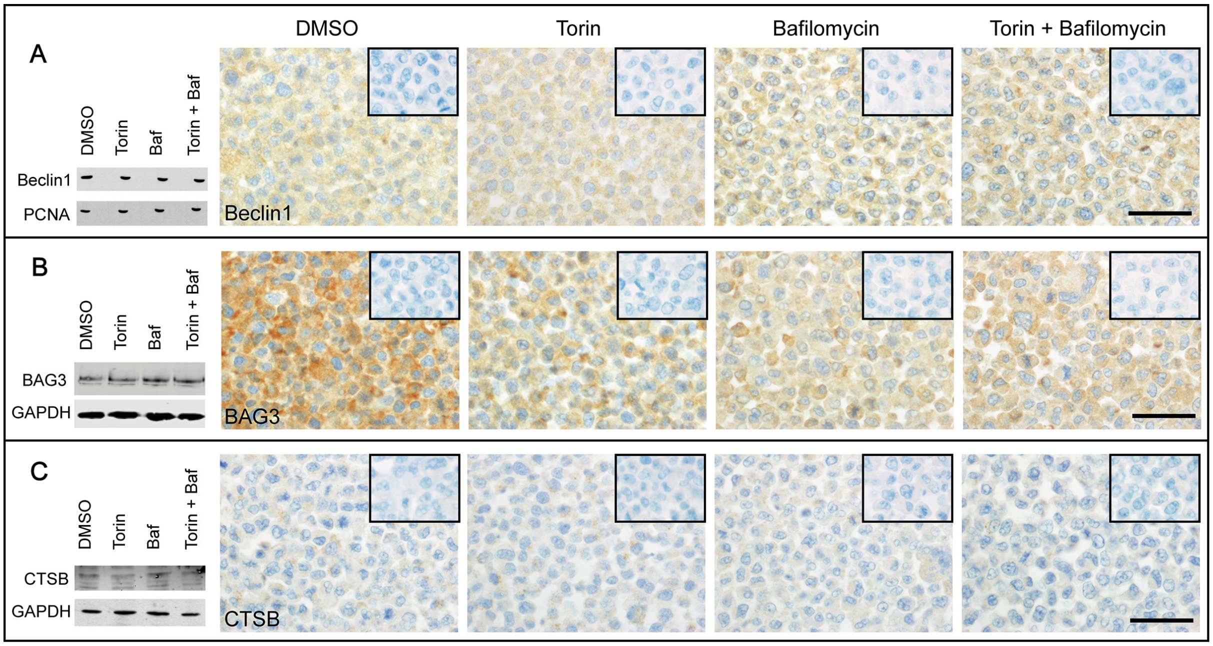

Supplementary Figure S1: Immunocytochemical analyses of Beclin1, BAG3 and CTSB. (**A–C**) Immunoblotting with anitbodies against Beclin1 (A), BAG3 (B) and Cathepsin B (CTSB) (C) of LNT-229 cells treated with DMSO as a control, Torin1 (250 nM), BafA1 (100 nM) or the combination treatment of both Torin1 and BafA1 (2 h each). Corresponding immunocytochemical analyses are depicted. Negative controls of the immunocytochemical stainings are provided in the right upper corner of the image (scale bars: 50 µm).

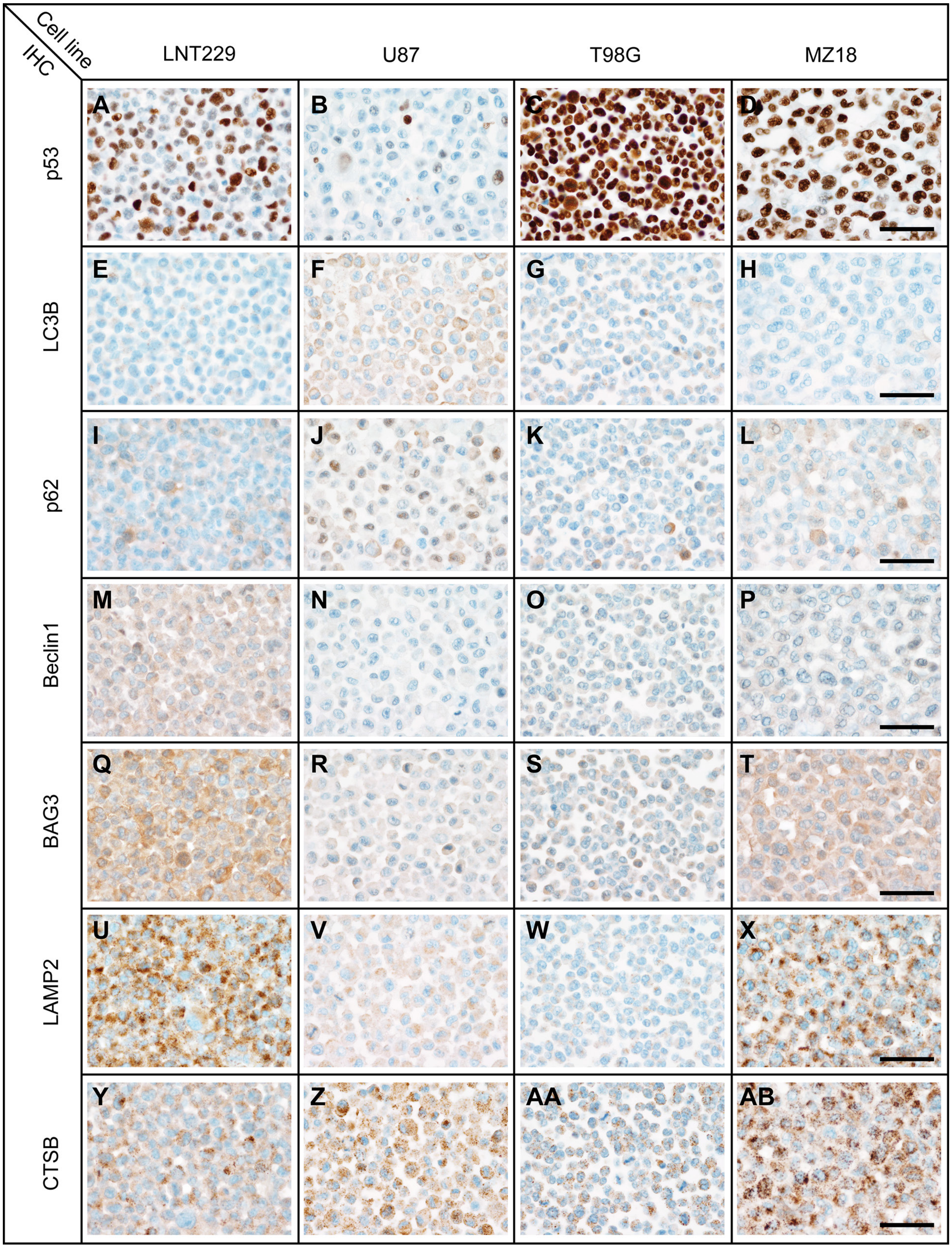

Supplementary Figure S2: p53 and ALN protein expression in glioma cell lines. (**A–D**) p53 immunocytochemistry of LNT-229 (A), U87 (B), T98G (C) and MZ-18 (D), (scale bar: 50 µm); (**E–H**) LC3B immunocytochemistry of LNT-229 (E), U87 (F), T98G (G) and MZ-18 (H), (scale bar: 50 µm); (**I–L**) p62 immunocytochemistry of LNT-229 (I), U87 (J), T98G (K) and MZ-18 (L), (scale bar: 50 µm); (**M–P**) Beclin1 immunocytochemistry of LNT-229 (M), U87 (N), T98G (O) and MZ-18 (P), (scale bar: 50 µm); (**Q–T**) BAG3 immunocytochemistry of LNT-229 (Q), U87 (R), T98G (S) and MZ-18 (T), (scale bar: 50 µm); (**U–X**) LAMP2 immunocytochemistry of LNT-229 (U), U87 (V), T98G (W) and MZ-18 (X), (scale bar: 50 µm); (**Y–AB**) CTSB immunocytochemistry of LNT-229 (Y), U87 (Z), T98G (AA) and MZ-18 (AB), (scale bar: 50 µm).

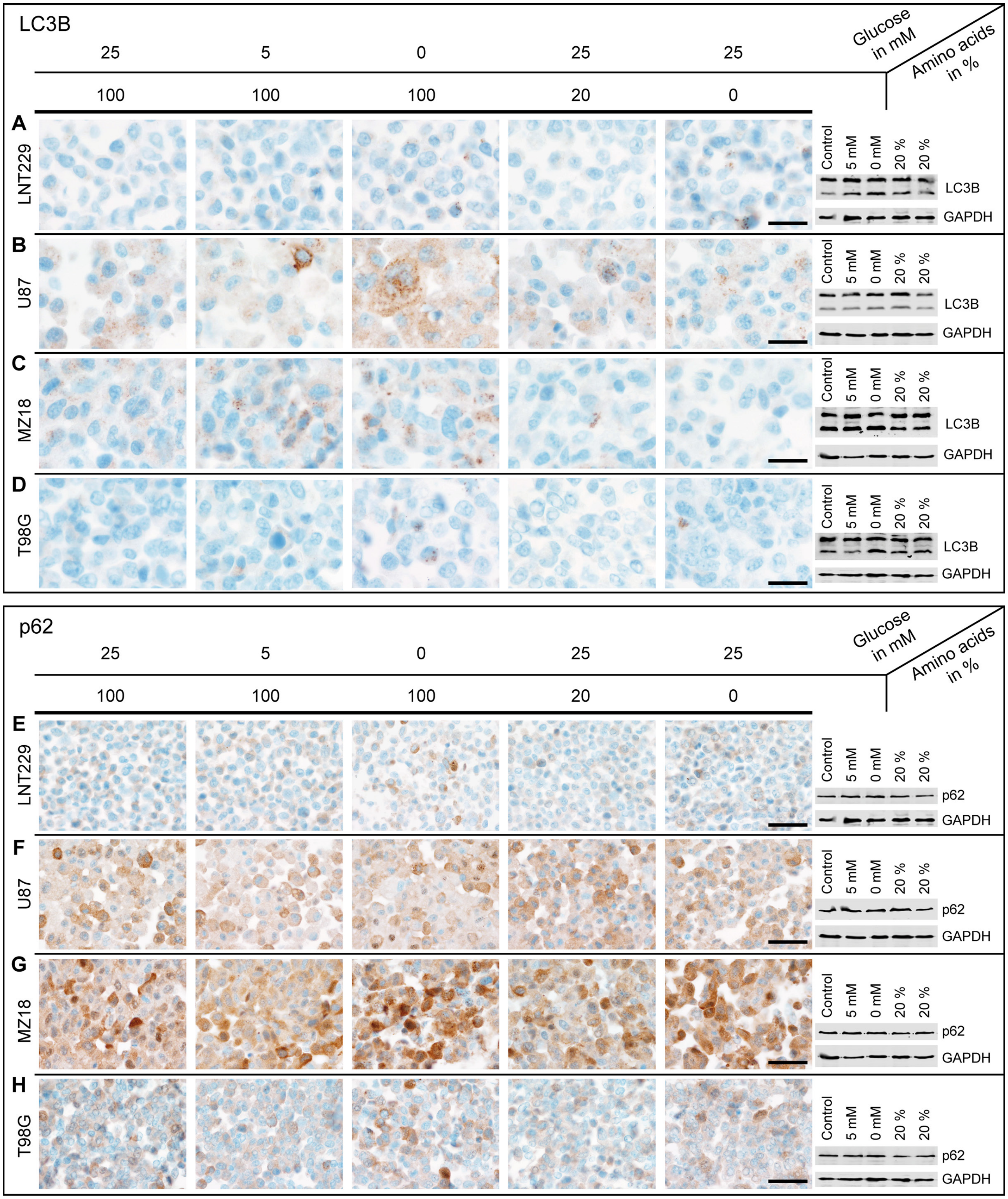

Supplementary Figure S3: LC3B and p62 analysis of starved glioma cell lines with different p53 status. (**A–D**) From left to right: LC3B immunocytochemistry of LNT-229 (p53 wild-type) (A), U87 (p53 wild-type) (B), MZ-18 (mutated p53) (C) and T98G (mutated p53) (D) cells in control condition, with only 5 mM or 0 mM glucose and deprived of the amino acids lysine, arginine and glutamine with only 20% or 0% of the content of the control condition (scale bar: 25 µm). Corresponding LC3B western blots of the same cell culture conditions are depicted. (**E–H**) From left to right: p62 immunocytochemistry of LNT-229 (A), U87 (B), MZ-18 (C) and T98G (D) cells in control condition, with only 5 mM or 0 mM glucose and deprived of the amino acids lysine, arginine and glutamine with only 20% or 0% of the content of the control condition (scale bar: 50 µm). Corresponding p62 western blots of the same cell culture conditions are depicted.

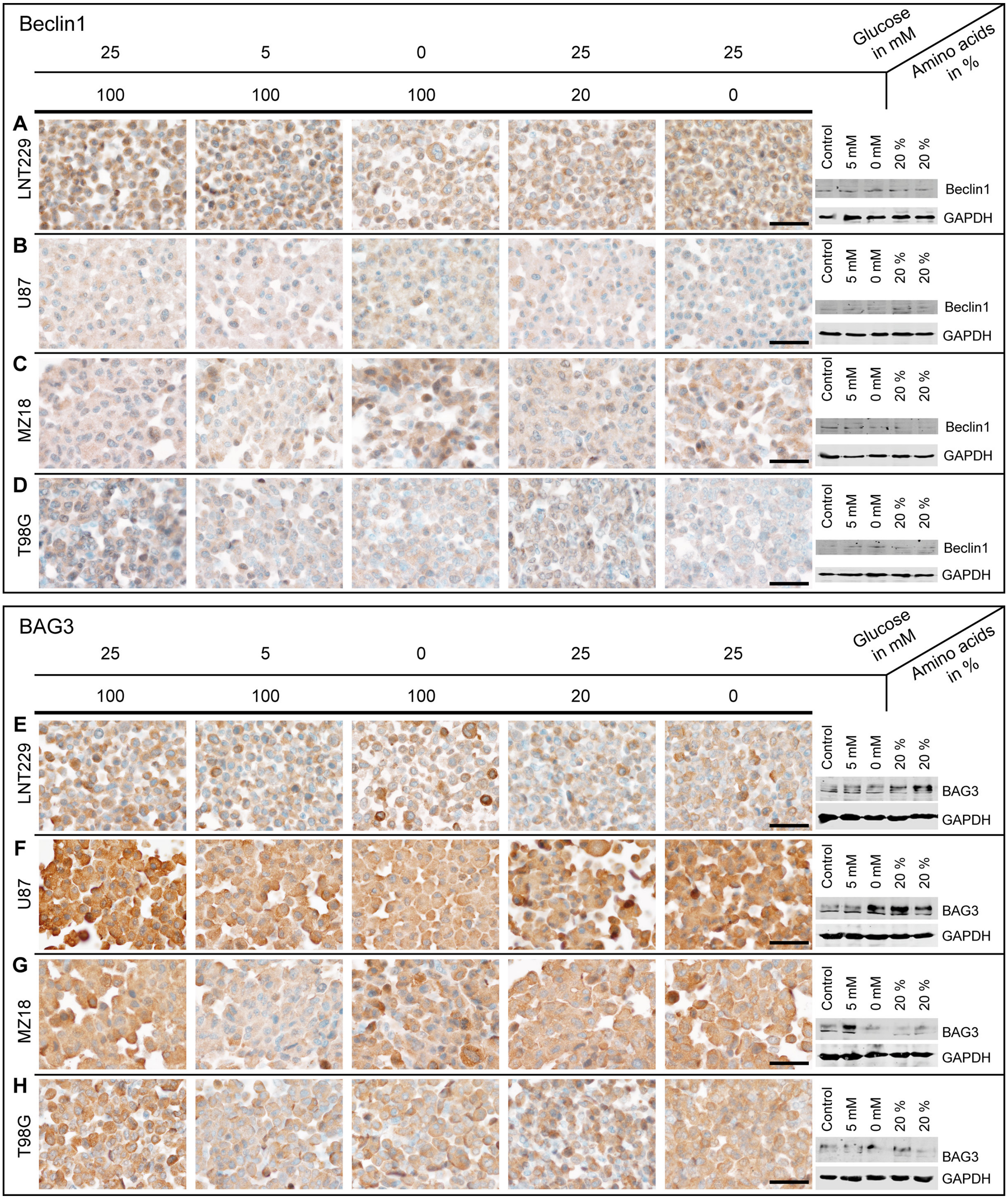

Supplementary Figure S4: Beclin1 and BAG3 analysis of starved glioma cell lines with different p53 status. (**A–D**) From left to right: Beclin1 immunocytochemistry of LNT-229 (A), U87 (B), MZ-18 (C) and T98G (D) cells in control condition, with only 5 mM or 0 mM glucose and deprived of the amino acids lysine, arginine and glutamine with only 20% or 0% of the content of the control condition (scale bar: 50 µm). Corresponding Beclin1 western blots of the same cell culture conditions are depicted. (**E–H**) From left to right: BAG3 immunocytochemistry of LNT-229 (A), U87 (B), MZ-18 (C) and T98G (D) cells in control condition, with only 5 mM or 0 mM glucose and deprived of the amino acids lysine, arginine and glutamine with only 20% or 0% of the content of the control condition (scale bar: 50 µm). Corresponding BAG3 western blots of the same cell culture conditions are depicted.

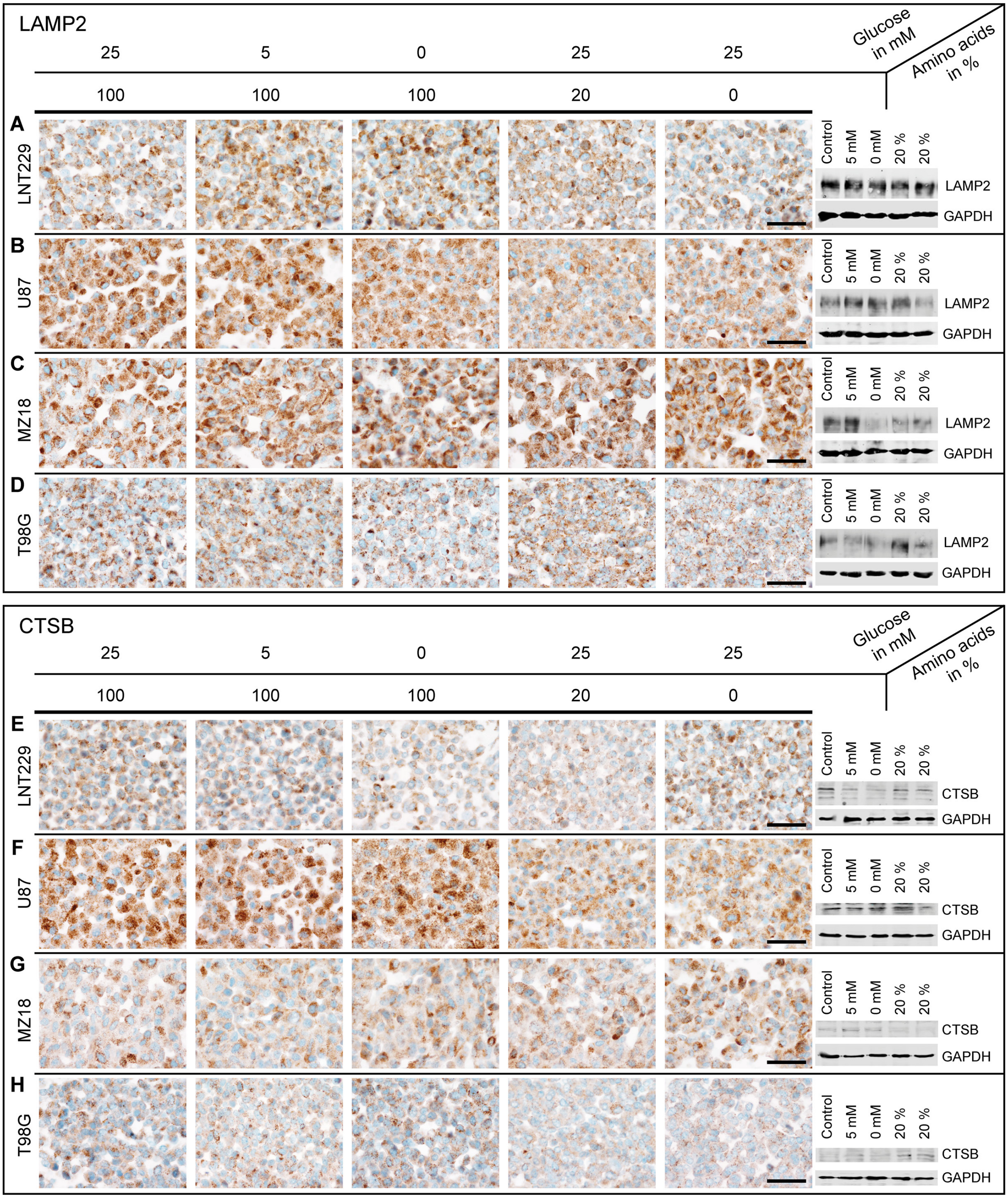

Supplementary Figure S5:LAMP2 and CTSB analysis of starved cell lines with different p53 status. (**A–D**) From left to right: LAMP2 immunocytochemistry of LNT-229 (A), U87 (B), MZ-18 (C) and T98G (D) cells in control condition, with only 5 mM or 0 mM glucose and deprived of the amino acids lysine, arginine and glutamine with only 20% or 0% of the content of the control condition (scale bar: 50 µm). Corresponding LAMP2 western blots of the same cell culture conditions are depicted. (**E–H**) From left to right: CTSB immunocytochemistry of LNT-229 (A), U87 (B), MZ-18 (C) and T98G (D) cells in control condition, with only 5 mM or 0 mM glucose and deprived of the amino acids lysine, arginine and glutamine with only 20% or 0% of the content of the control condition (scale bar: 50 µm). Correspondings CTSB western blots of the same cell culture conditions are depicted.

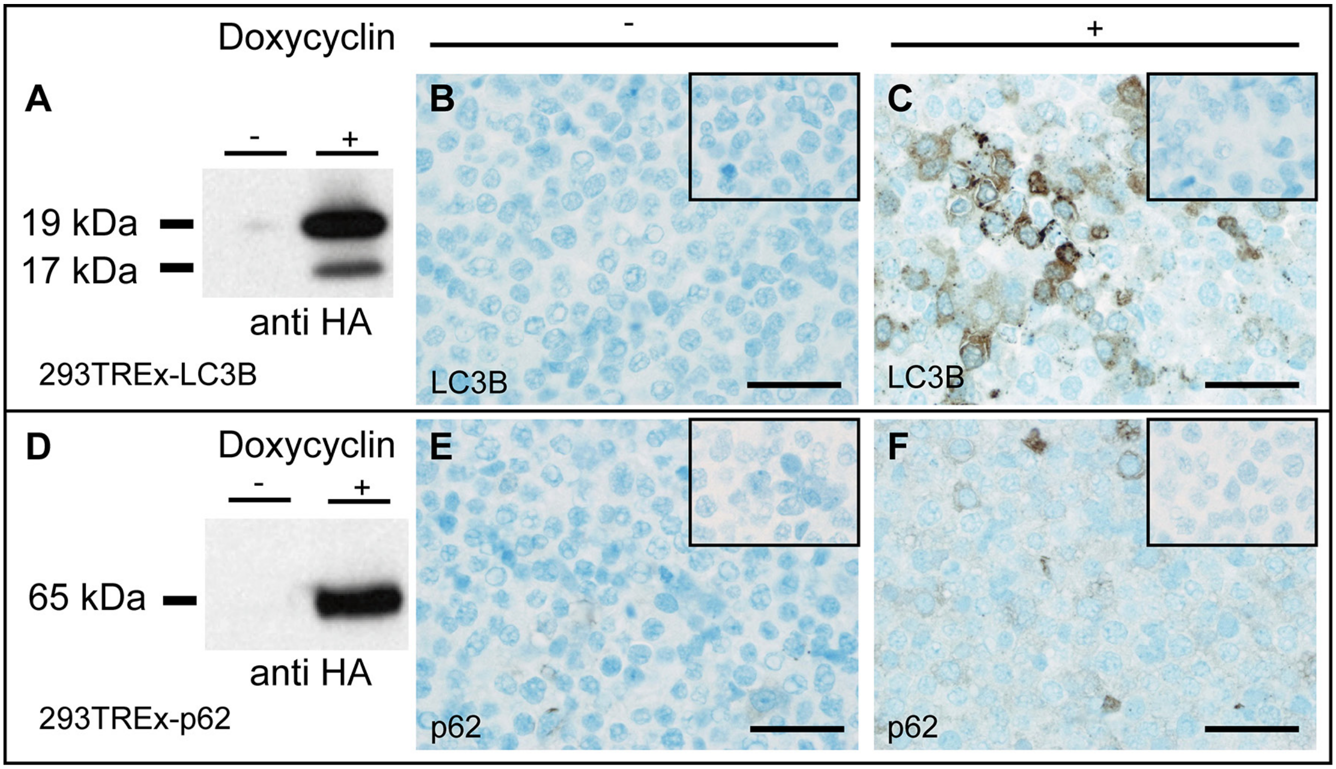

Supplementary Figure S6: Doxycycline-inducible expression of LC3B and p62. (**A**) Immunoblotting against the HA-tag after doxycycline-induced expression of HA-tagged LC3B (**A**) and p62 (**D**) and corresponding immunocytochemical staining of LNT-229 glioma cells treated (**B**/**E**) without or (**C**/**F**) with Doxycycline.

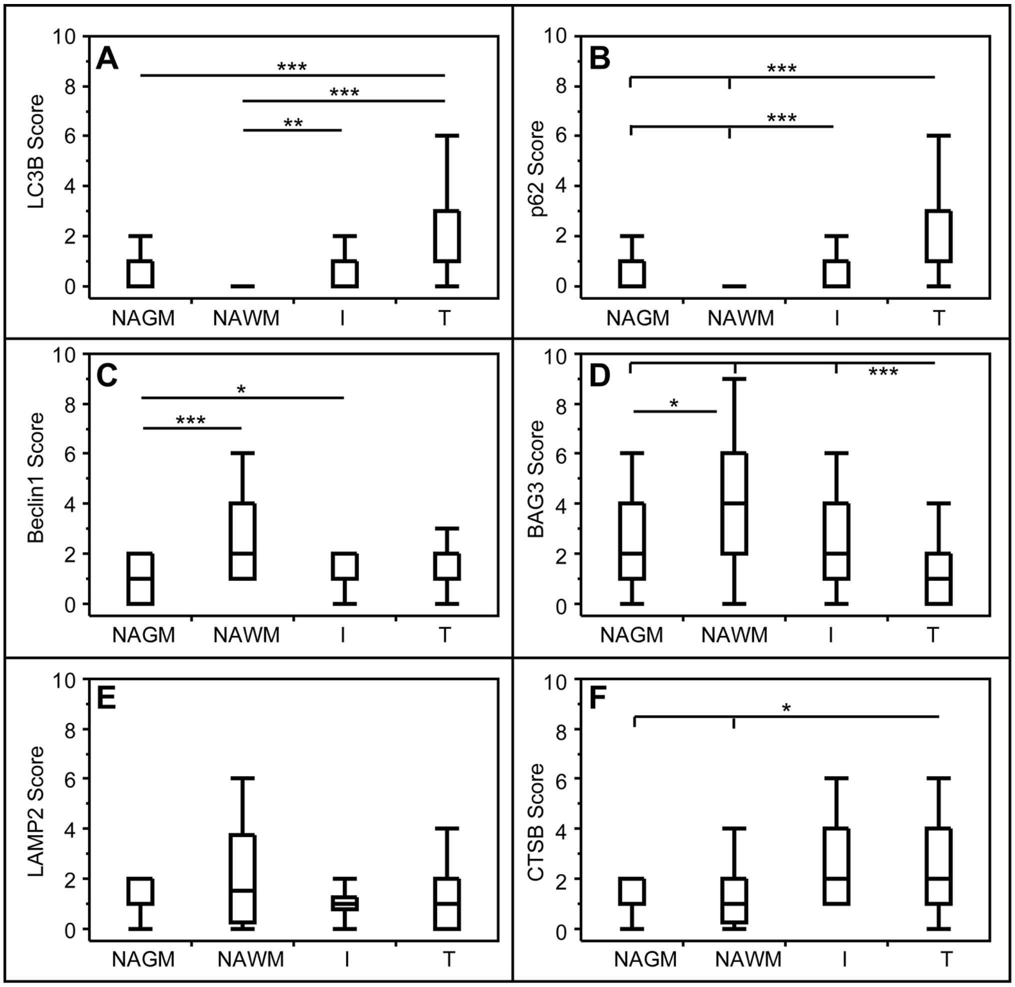

Supplementary Figure S7: Autophagy-associated and lysosomal markers in different areas of glioblastoma. (**A**) Box blots showing LC3B immunohistochemical scores of glioblastomas WHO grade IV from 44 normal appearing grey matter (NAGM) (min: 0, max: 2, median: 0), 17 normal appearing white matter (NAWM) (min: 0, max: 1, median: 0), 35 infiltration zone (I) (min: 0, max: 6, median: 1) and 73 tumor center (T) (min: 0, max: 9, median: 1). (**B**) Box blots showing p62 immunohistochemical scores of glioblastomas WHO grade IV from 46 NAGM (min: 0, max: 2, median: 0), 18 NAWM (min: 0, max: 1, median: 0), 38 I (min: 0, max: 4, median: 0.5) and 75 T (min: 0, max: 4, median: 1). (**C**) Box blots showing Beclin1 immunohistochemical scores of glioblastomas WHO grad IV from 47 NAGM (min: 0, max: 2, median: 1), 15 NAWM (min: 1, max: 6, median: 2), 39 I (min: 0, max: 9, median: 1) and 74 T (min: 0, max: 6, median: 1). (**D**) Box blots showing BAG3 immunohistochemical scores of glioblastomas WHO grade IV from 47 NAGM (min: 0, max: 6, median: 2), 18 NAWM (min: 0, max: 9, median: 4), 38 I (min: 0, max: 6, median: 2) and 71 T (min: 0, max: 6, median: 1). (**E**) Box blots showing LAMP2 immunohistochemical scores of glioblastomas WHO grade IV from 47 NAGM (min: 0, max: 6, median: 1), 16 NAWM (min: 0, max: 6, median: 1.5), 38 I (min: 0, max: 4, median: 1) and 73 T (min: 0, max: 12, median: 1). (**F**) Box blots showing CTSB immunohistochemical scores of glioblastomas WHO grade IV from 46 NAGM (min: 0, max: 6, median: 2), 16 NAWM (min: 0, max: 4, median: 1), 38 I (min: 1, max: 9, median: 2) and 74 T (min: 0, max: 12, median: 2).

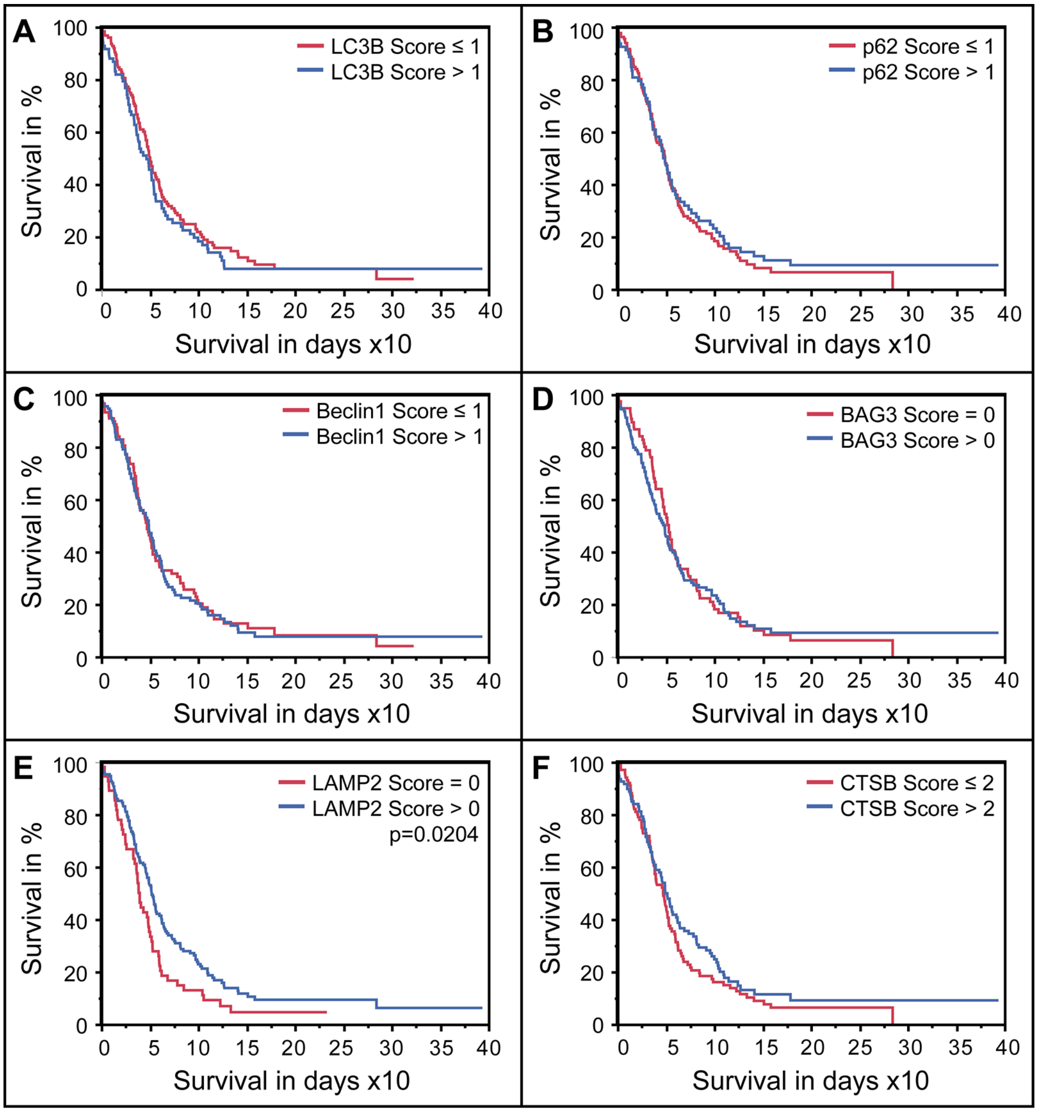

Supplementary Figure S8: Survival analyses of glioblastoma patients related to autophago-lysosomal pathway activation status. (**A**) Kaplan-Meyer analysis of 177 glioblastoma patients stratified according to immunohistochemical LC3B scores of 0 and 1 (n = 107, median survival: 503 days) and more than 1 (n = 70, median survival: 465 days). (**B**) Kaplan-Meyer analysis of 180 glioblastoma patients stratified according to immunohistochemical p62 scores of 0 and 1 (n = 114, median survival: 490 days) and more than 1 (n = 66, median survival: 489 days). (**C**) Kaplan-Meyer analysis of 169 glioblastoma patients stratified according to immunohistochemical Beclin1 scores of 0 and 1 (n = 75, median survival: 470 days) and more than 1 (n = 94, median survival: 489 days). (**D**) Kaplan-Meyer analysis of 170 glioblastoma patients stratified according to immunohistochemical BAG3 scores of 0 (n = 68, median survival: 529 days) and more than 0 (n = 102, median survival: 482 days); (**E**) Kaplan-Meyer analysis of 174 glioblastoma patients stratified according to immunohistochemical LAMP2 scores of 0 (n = 51, median survival: 394 days) and more than 0 (n = 123, median survival: 489 days) Wilcoxon: p = 0.0204. (**F**) Kaplan-Meyer analysis of 177 glioblastoma patients stratified according to immunohistochemical BAG3 scores of 0–2 (n = 90, median survival: 472 days) and more than 2 (n = 87, median survival: 492 days).

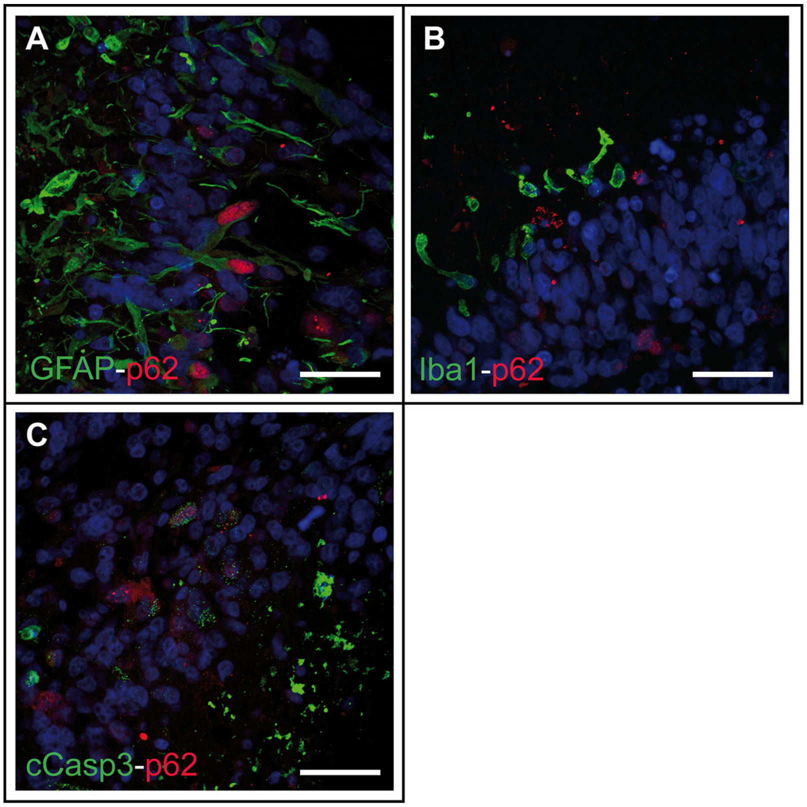

Supplementary Figure S9: Glioma cells are the major source of p62 expression in glioblastoma. Double immunofluorescent staining in glioblastoma against p62 and (**A**) GFAP, (**B**) Iba1, (**C**) cCasp3 (scale bars: 50 µm).

Supplementary Table S1: Cytopellet micro array conditions

Cell line	Rapamycin in nM	Torin in nM	Hif1-α Knockdown	Oxygen in %	Glucose in mM	Glutamine in mM	Duration in h
LNT-229	0	0	No construct	21	25	4	24
LNT-229	0	0	No construct	21	25	4	24
LNT-229	0	100	No construct	21	25	4	24
LNT-229	0	0	No construct	21	25	4	48
LNT-229	100	0	No construct	21	25	4	48
LNT-229	0	100	No construct	21	25	4	48
LNT-229	0	0	No construct	21	0	4	24
LNT-229	100	0	No construct	21	0	4	24
LNT-229	0	100	No construct	21	0	4	24
LNT-229	0	0	No construct	21	0	4	48
LNT-229	100	0	No construct	21	0	4	48
LNT-229	0	100	No construct	21	0	4	48
LN-229	0	0	knockdown	1	0	4	24
LN-229	0	0	knockdown	21	0	0	24
LN-229	0	0	knockdown	21	0	4	24
LN-229	0	0	KTR	21	0	0	24
LN-229	0	0	KTR	1	25	4	24
LN-229	0	0	knockdown	1	25	4	24
LN-229	0	0	knockdown	1	0	0	24
LN-229	0	0	KTR	1	0	4	24
LN-229	0	0	knockdown	21	25	4	24
LN-229	0	0	KTR	21	0	4	24
LN-229	0	0	KTR	1	0	0	24
LN-229	0	0	KTR	21	25	4	24
LN-229	0	0	KTR	21	25	4	48
LN-229	0	0	knockdown	21	0	4	48
LN-229	0	0	knockdown	21	25	4	48
LN-229	0	0	KTR	21	0	4	48
